# Pathological calcium overload impairs skeletal muscle regeneration through sustained NFAT-dependent proliferative signaling

**DOI:** 10.1038/s12276-026-01856-3

**Published:** 2026-10-02

**Authors:** Sangsoo Lee, Namjoon Cho, Seokwoo Jo, Jong-Hun Ji, Jong Ok Kim, Chungoo Park, Sunkyung Choi, Eun-Mi Kim, Kee K. Kim

**Affiliations:** 1 Department of Biochemistry, College of Natural Sciences, Chungnam National University, Daejeon, Republic of Korea; 2 School of Biological Science and Technology, Chonnam National University, Gwangju, Republic of Korea; 3 Department of Orthopedic Surgery, Daejeon St. Mary’s Hospital, The Catholic University of Korea, Daejeon, Republic of Korea; 4 Department of Pathology, Daejeon St. Mary’s Hospital, The Catholic University of Korea, Daejeon, Republic of Korea; 5 Department of Biological Sciences, College of Natural Sciences, Keimyung University, Daegu, Republic of Korea; 6 Department of Bio and Environmental Technology, Seoul Women’s University, Seoul, Republic of Korea

**Keywords:** Calcium signalling, Differentiation, Experimental models of disease, Translational research, Diagnostic markers

## Abstract

Skeletal muscle regeneration requires coordinated myoblast proliferation and differentiation. Calcium dysregulation is commonly observed in aging and disease-associated muscle weakness; however, its impact on myogenic progression remains unclear. In this study, we investigated the mechanism by which intracellular calcium overload impairs myogenic differentiation and muscle regeneration. Transcriptomic analysis of aged human muscle revealed coordinated activation of calcium signaling and proliferation pathways. In vitro calcium exposure increased myoblast proliferation under differentiation conditions while suppressing myogenic markers. RNA sequencing identified upregulation of proliferative programs with concurrent suppression of myogenic genes and enrichment of calcium–NFAT signaling. Mechanistically, calcium promoted NFATc1 nuclear translocation and upregulated *Ccnd1* expression. Consistent with these in vitro findings, both ovariectomized mice (systemic calcium dysregulation) and human calcific tendinitis tissues (localized calcium overload) exhibited NFAT-associated proliferative signatures with suppressed myogenic markers. The selective NFAT inhibitor VIVIT prevented calcium-induced NFATc1 nuclear accumulation and restored myotube formation in vitro, while promoting myofiber maturation in an in vivo injury model. These findings implicate chronic calcium–NFAT signaling as a mechanism linking calcium dysregulation to impaired muscle regeneration.

## Myoblast proliferation linked to calcium overload

Skeletal muscle regeneration relies on a precise balance between cell proliferation and differentiation, a process disrupted by calcium dysregulation. This study reveals that chronic calcium overload impairs myogenesis by sustaining nuclear factor of activated T cells (NFAT)-dependent proliferative signaling, preventing myogenic commitment. Researchers conducted transcriptomic analyses of aged human muscle, mechanistic studies in calcium-exposed myoblasts, and validation in an ovariectomy mouse model. They found that elevated calcium levels lock myoblasts in a proliferative state through NFATc1 nuclear translocation and cyclin D1 (CCND1) upregulation. Importantly, pharmacological NFAT inhibition with the VIVIT peptide restored myogenesis under calcium overload, highlighting the calcium–NFAT axis as a potential therapeutic target for muscle weakness. These findings suggest that targeting NFAT signaling could enhance muscle regeneration in conditions characterized by calcium dysregulation, offering a promising avenue for future research and therapeutic development.

This summary was initially drafted using artificial intelligence, then revised and fact-checked by the author.

## Introduction

Skeletal muscle regeneration is orchestrated through a tightly ordered program that begins with the activation of satellite cells. Once activated, these quiescent progenitors proliferate, expand clonally as myoblasts, and then exit the cell cycle. Subsequently, they fuse into multinucleated myotubes that mature into contractile muscle fibers^1–3^. This transition is marked by the suppression of proliferative programs that include PAX7 and cyclin D1 (CCND1) and by the induction of myogenic transcriptional machinery and structural proteins such as members of the myosin heavy chain (MYH) family^4,5^. Successful muscle regeneration therefore requires precise temporal coordination between the termination of proliferation and myogenesis. These decisions are regulated by integrated signaling networks, including the calcium ion (Ca^2+^) signaling pathway, which determine whether progenitors continue to proliferate, return to a quiescent state through self-renewal, or commit to terminal differentiation. Dysregulation of cell-cycle exit or disruption of the myogenic program can disturb muscle fiber formation, ultimately imparing muscle tissue repair.

Calcium ions serve as a ubiquitous second messenger, coupling signals from the cell surface and organelles to effectors in the cytoplasm and nucleus across mutiple cell types^6–8^. Properly regulated Ca^2+^ homeostasis is essential for metabolism, signal transduction, and gene expression, and is maintained by Ca^2+^-responsive pathways^9–11^. A variety of clinical conditions disrupt this balance and cause chronically elevated Ca^2+^ levels. Conditions including endocrine hypercalcemia, such as primary hyperparathyroidism, parathyroid hormone-induced peptide, osteolytic malignancies, lymphomas with aberrant calcitriol production, immobility, and vitamin D intoxication, are substantially affected because of Ca^2+^ disruption^12–15^. Proximal muscle weakness is a common clinical accompaniment in these states. Systemic hypercalcemia, defined by total serum Ca^2+^ levels exceeding 3.0 mM, exposes skeletal muscle to chronic Ca^2+^ overload and is clinically associated with generalized fatigue and proximal muscle weakness^16^. In addition, Duchenne muscular dystrophy causes chronic cytosolic Ca^2+^ overload because of dystrophin loss, and Ca^2+^ dysregulation is known as a major pathogenic axis^17,18^. Aging provides an additional physiological context in which intracellular Ca^2+^ levels increase, Ca^2+^ handling becomes inefficient, and sarcopenic muscle undergoes aberrant Ca^2+^ signaling pathway^19,20^. These clinical observations connect Ca^2+^ overload to muscle weakness. Despite accumulating evidence supporting the link between Ca^2+^ elevation and muscle weakness, research on the role of Ca^2+^ in muscle has primarily focused on Ca^2+^-mediated excitation–contraction coupling in mature muscle fibers^21–23^.

Although the transition from progenitor cells to myotubes is crucial for maintaining the adult musculoskeletal system, Ca^2+^ signaling cascades influence myogenesis by regulating satellite cell self-renewal, proliferation, and differentiation^24–27^. Aberrant Ca^2+^ signaling can remodel channel activity, alter organelle function, and induce maladaptive transcriptional programs in skeletal muscle. Previous studies suggest that Ca^2+^ signaling in skeletal muscle regulates myogenesis, including the control of fiber-type specification and hypertrophy in mature muscle through the Ca^2+^-mediated nuclear factor of activated T cells (NFAT) signaling pathway^28,29^. However, the mechanistic link between pathological Ca^2+^ overload and muscle weakness remains unclear.

Here, we demonstrate that chronically elevated Ca^2+^ levels impair myogenesis by sustaining NFAT-dependent proliferative signaling and preventing myogenic commitment. We integrate transcriptomic analysis of aged human skeletal muscle, mechanistic studies in Ca^2+^-exposed myoblasts, validation in an ovariectomy (OVX) mouse model exhibiting Ca^2+^ dysregulation, and examination of patient-derived calcified muscle tissues. Using the barium chloride (BaCl₂)-induced muscle injury model, we further demonstrate that pharmacological NFAT inhibition rescues muscle regeneration defects in vivo. Our findings reveal that pathological Ca^2+^ overload locks myoblasts in a proliferative state through NFATc1 nuclear translocation and *CCND1* upregulation, thereby blocking myogenesis. Furthermore, pharmacological NFAT inhibition with VIVIT rescues myogenesis under Ca^2+^ overload, identifying the Ca^2+^–NFAT axis as a potential therapeutic target for muscle weakness associated with Ca^2+^ dysregulation.

## Materials and methods

### Human specimens

Supraspinatus muscle tissues were obtained by arthroscopic punch during calcific deposit removal from patients with calcific tendinitis. Samples from areas adjacent to calcific deposits and remote regions were isolated for analysis. All patients provided informed consent, and the study was approved by the Institutional Review Board (IRB) of the Catholic University of Korea (IRB No. DC23TASI0063).

### Analysis of public human gastrocnemius muscle transcriptome data

Human gastrocnemius muscle transcriptomes across age groups were obtained from GTEx v8 (https://gtexportal.org) including data from 679 individuals aged≥ 50 or < 40 years^30^. Gene set enrichment analysis (GSEA) was performed to identify age-associated transcriptomic changes. Gene sets with an absolute normalized enrichment score |NES| > 1 and adjusted *P*-value < 0.05 were considered significant.

### Animal models

To establish an OVX mouse model, female ICR mice (6 weeks old; *n* = 5 per group) were obtained from Daehan Biolink and housed under controlled conditions (22 ± 2 °C, 40–60% humidity, 12-h light–dark cycle). After 1 week of acclimatization, mice were ovariectomized under anesthesia by injecting 250 mg/kg 2,2,2-tribromoethanol intraperitoneally to induce osteoporosis. Muscle endurance was assessed after 5 weeks using a treadmill test. Mice ran at 10 cm/s for 3 min (0% incline), then the speed was increased to 15 cm/s and 50 cm/s until exhaustion, defined as the inability to maintain running for 10 s. At 6 weeks, mice were euthanized by CO₂ inhalation. Gastrocnemius muscles and femurs were collected. Femurs were subjected to micro-computed tomography (micro-CT) scanning using a Viva CT40 system (SCANCO Medical AG). Bone morphometric parameters, including total femoral volume (TV), bone surface-to-tissue volume ratio (BS/TV), and bone mineral density were quantified.

To assess the role of NFAT signaling in muscle regeneration in vivo, male ICR mice (5 weeks old, *n* = 5 per group) were subjected to acute skeletal muscle injury. Under anesthesia, both the tibialis anterior (TA) muscles were injected with 100 μL of 1.2% BaCl₂ (Sigma-Aldrich) dissolved in sterile saline to induce myofiber damage and regeneration. To test the therapeutic effect of NFAT inhibition, mice were injected with VIVIT peptide (170 μg in 100 μL sterile saline; 95% purity, synthesized and HPLC-confirmed by Peptron) intramuscularly into one TA muscle at day 0 (simultaneously with BaCl₂) and day 2 post-injury. The contralateral TA muscle received an equal volume of sterile saline at the same time points and served as an internal control. At 3 days post-injury, TA muscles were harvested, fixed in 4% paraformaldehyde, embedded in Optimal Cutting Temperature (O.C.T.) Compound (Tissue-Tek), and cryosectioned at 12-μm thickness for immunofluorescence analysis. All animal procedures were approved by the Institutional Animal Care and Use Committee of Chungnam National University (approval no. 202109A-CNU-142).

### Mouse myoblast cell culture and differentiation

C2C12 myoblasts were cultured in Dulbecco’s modified Eagle medium (DMEM; WELGENE) supplemented with 10% fetal bovine serum (Gibco) and 1% penicillin–streptomycin (WELGENE) at 37 °C in a humidified atmosphere containing 5% CO₂. At approximately 90% confluence, the medium was replaced with differentiation medium to induce myogenic differentiation.

Primary myoblasts were isolated from the hindlimb skeletal muscles of postnatal day 3 mice. Excised muscle tissues were trimmed to remove bone and adipose tissue and enzymatically digested in a 1:1 mixture of collagenase D (1.5 U/ml) and dispase (2.4 U/ml) for 1 h at 37 °C. Cell suspensions were collected by centrifugation (300 x *g* for 5 min) and resuspended in growth medium composed of Ham’s F-10 nutrient mixture (Gibco) supplemented with 10% calf serum (Gibco), 2.5 μg/ml recombinant bFGF, and 1% penicillin–streptomycin. Cells were seeded onto poly-L-ornithine (Sigma-Aldrich)-coated culture dishes and maintained in a humidified incubator at 37 °C with 5% CO₂. For myogenic differentiation, cells were plated on collagen type I-coated dishes (Santa Cruz Biotechnology), and at approximately 90% confluence, the medium was replaced with differentiation medium comprising DMEM supplemented with 2% horse serum and 1% penicillin–streptomycin.

### Differentiation of hESC-derived myoblasts

Human embryonic stem cell (hESC) line H9 was obtained from WiCell Research Institute. H9 cells were cultured on hESC-qualified Matrigel (Corning)-coated plates in mTeSR1 medium (STEMCELL Technologies) at 37 °C in a humidified incubator containing 5% CO_2_. To generate hESC-derived myoblasts, cells were cultured for 7 days in a Di-CL medium comprising DMEM/F12 supplemented with 1% ITS, 1% nonessential amino acids (NEAA), 0.2% penicillin–streptomycin, 3 μM CHIR-99021, 0.5 μM LDN-193189, and 20 ng/ml recombinant FGF2. Cells were then cultured for 3 weeks in DK-HIFL medium, composed of DMEM/F12 supplemented with 15% knockout serum replacement, 1% NEAA, 0.2% penicillin–streptomycin, 0.1 mM 2-mercaptoethanol, 10 ng/ml recombinant HGF, 2 ng/ml recombinant IGF1, 20 ng/ml recombinant FGF2, and 0.5 μM LDN-193189, to induce differentiation into hESC-derived myoblasts. Cells were subsequently maintained in skeletal muscle cell growth medium (SkGM; Lonza) for 2 weeks. For myogenic differentiation, hESC-derived myoblasts were cultured in DK-HI medium containing DMEM/F12 supplemented with 15% knockout serum replacement, 1% NEAA, 0.2% penicillin–streptomycin, 0.1 mM 2-mercaptoethanol, 10 ng/ml recombinant HGF, and 2 ng/ml recombinant IGF1 for 2 weeks^31^. The study was approved by the Public Institutional Review Board of the Korea Ministry of Health and Welfare (approval No. P01-202504-03-001).

### RNA-seq and data analysis

RNA quality was assessed using the Bioanalyzer 2100 system (Agilent Technologies), and 1 µg of high-quality RNA was used to prepare sequencing libraries with the TruSeq Stranded mRNA LT Sample Prep Kit (Illumina). Libraries were sequenced on an Illumina NovaSeq platform. Low-quality bases and adapter sequences were removed using Trimmomatic (v0.36)^32^. Clean reads were aligned to the mouse reference genome (GRCm39) using HISAT2 (v.2.2.1) with default parameters, and gene expression levels were quantified using Cufflinks (v2.1.1)^33,34^. Genes with fragments per kilobase of transcript per million mapped reads (FPKM) values < 0.01 in any sample were excluded from downstream analyses. Differentially expressed genes (DEGs) were analyzed for functional enrichment using Database for Annotation, Visualization, and Integrated Discovery (DAVID; https://david.ncifcrf.gov/) and GSEA v4.3.2^35,36^. Gene sets with |NES| > 1 and adjusted *P*-value< 0.05 were considered significant.

### Cell viability assays

Myoblast viability after exposure was assessed using propidium iodide (PI) staining. Differentiated myotubes were incubated with 25 µg/ml PI in phosphate-buffered saline (PBS) for 15 min at room temperature without fixation, and PI-positive cells were quantified using fluorescence microscopy. To establish a reference baseline for total cell numbers, total nuclei were visualized in parallel cultures fixed in 4% paraformaldehyde and permeabilized with 0.1% Triton X-100 before PI staining. Fluorescence images were acquired using a fluorescence microscope (pE-300 lite, Meiji Techno).

### Immunoblot analysis

Protein extracts were prepared from myoblasts using a Tris-based lysis buffer with protease inhibitors (Roche). Equal amounts of protein were separated via sodium dodecyl sulfate (SDS)–polyacrylamide gel electrophoresis and transferred to nitrocellulose membranes (Pall Corporation). Membranes were blocked with 5% nonfat dried milk in PBS containing 0.1% Tween-20 (PBST) for 1 h and then incubated overnight at 4 °C with primary antibodies targeting MYH3 (sc-53091), MYH (sc-376157), MYOG (sc-52903), MYOD (sc-377460), PAX7 (sc-81648), and GAPDH (sc-47724), all procured from Santa Cruz Biotechnology. After washing with PBST, membranes were incubated with HRP-conjugated secondary antibodies (Abcam). Protein bands were visualized using enhanced chemiluminescence reagents (Thermo Fisher Scientific) and quantified with ImageJ software.

### RNA extraction and qRT-PCR

Total RNA was isolated from the gastrocnemius muscles of animal models and from mouse myoblasts using the Hybrid-R kit (GeneAll). cDNA was synthesized from 1 µg of RNA using M-MLV reverse transcriptase (Promega), random hexamer primers, and an RNase inhibitor (Enzynomics). Quantitative PCR (qPCR) was performed using SYBR Green (GENETBIO) on the AriaMx Real-Time PCR System (Agilent Technologies). Primer sequences are listed in Supplementary Table 1.

### Intracellular Ca^2+^ imaging

Differentiated myotubes were washed with Dulbecco's PBS and incubated with 1 µM Fluo-4 AM (Invitrogen) at 37 °C for 30 min, followed by incubation for 30 min at 25 °C in the dark for de-esterification. Fluorescence images were acquired using a fluorescence microscope. Intracellular Ca^2+^ levels were quantified based on fluorescence intensity with ImageJ software.

### Immunofluorescence analysis

Myoblasts were fixed in 4% paraformaldehyde for 10 min and permeabilized with 0.5% Triton X-100 in PBS for 15 min. Mouse tissue samples were fixed in 4% paraformaldehyde solution for 24 h, immersed in a 30% sucrose solution for 16 h, and then embedded in Tissue-Tek O.C.T. Compound (Sakura Finetek) to prepare frozen sections. The frozen tissues were sectioned at a thickness of 12 μm.

All samples were blocked by incubating with 5% goat serum and 0.1% bovine serum albumin for 1 h, and stained with primary antibodies against MYH3 (sc-53091, Santa Cruz Biotechnology), MYH (sc-376157), NFATc1 (sc-17834), and GFP (sc-9996). After washing the slides, they were incubated with Alexa Fluor 488- and 594-conjugated goat anti-mouse IgG (Thermo Fisher Scientific). Nuclei were counterstained with DAPI (Sigma-Aldrich), and samples were mounted using ProLong Gold Antifade Mountant (Thermo Fisher Scientific). Fluorescence images were acquired using a Zeiss LSM 880 confocal laser-scanning microscope (Carl Zeiss, Germany). Fluorescence intensity was quantified with ImageJ software.

### NFATc1-GFP translocation assay

C2C12 cells and primary mouse myoblasts were transfected with NFATc1-GFP (24219; Addgene). Transfection was performed using Lipofectamine Stem Transfection Reagent (Invitrogen) according to the manufacturer’s instructions. After differentiation under Ca^2+^ exposure, in the presence or absence of the NFAT inhibitor VIVIT, nuclear translocation was assessed by immunofluorescence and quantified using the nuclear-to-cytoplasmic intensity ratio.

### ChIP assay

Chromatin immunoprecipitation (ChIP) assays were performed using a ChIP Express kit (Millipore Biotechnology) according to the manufacturer’s instructions. Briefly, C2C12 cells were cross-linked for 5 min at 37 °C using 0.5% formaldehyde and lysed in SDS lysis buffer (50 mM Tris-HCl pH 8.0, 10 mM EDTA, and 1% SDS). Chromatin was then fragmented to an average size of 300–600 bp by sonication. After removing cellular debris, the chromatin was diluted fivefold in ChIP dilution buffer (16.7 mM Tris-HCl pH 8.0, 167 mM NaCl, 1.2 mM EDTA, 1.1% Triton X-100, and 0.01% SDS). The chromatin-containing supernatant was incubated with 2 μg of NFATc1-specific antibody (sc-17834, Santa Cruz Biotechnology) for 3 h at 4 °C with rotation. Immune complexes were subsequently captured with Dynabeads Protein G (Invitrogen) and rotated for an additional 2 h. To eliminate nonspecific binding, the magnetic beads were washed twice with each of the following buffers: low-salt, high-salt, lithium chloride, and TE. Bound DNA–protein complexes were then eluted in elution buffer consisting of 1% SDS and 0.1 M NaHCO₃. To reverse formaldehyde-induced crosslinks, the eluted fractions were supplemented with 200 mM NaCl and 200 μg/ml RNase A, followed by overnight incubation at 65 °C. Residual proteins were digested with 10 mM EDTA and 100 μg/ml Proteinase K for 1 h at 60 °C. The genomic DNA fragments were purified and used as templates for PCR to quantify the enrichment of NFATc1 at the Ccnd1 promoter. Band intensities were quantified by densitometric analysis with ImageJ software. Primer sequences are listed in Supplementary Table 1.

### Statistical analysis

Data are expressed as mean ± s.e.m. for bar graphs. For box plots, data are shown as median with 25th and 75th percentiles (quartiles) and minimum and maximum values. Statistical comparisons among groups were performed using one-way analysis of variance (ANOVA) followed by Tukey’s post hoc test, whereas an unpaired Student’s *t*-test was applied to determine statistical significance between two groups. Statistical significance was set at *P* < 0.05.

## Results

### Activation of Ca^2+^ signaling and proliferative pathways in aged skeletal muscle tissue

**Fig. 1:**
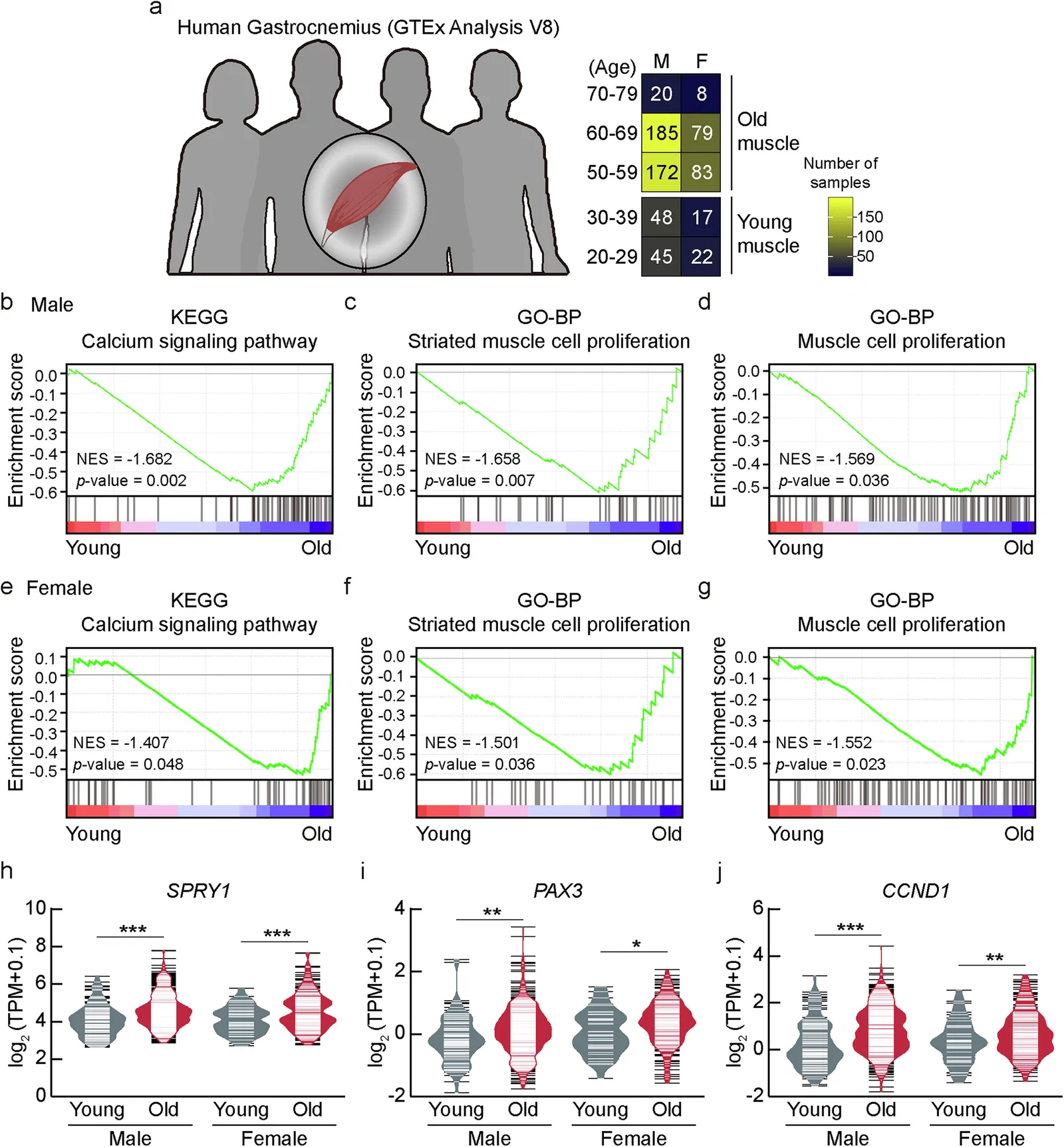
Aged skeletal muscle exhibits upregulation of Ca^2+^ signaling and proliferative pathways. **a** Age- and sex-specific distribution of transcriptome samples from human gastrocnemius muscle in the GTEx v8 dataset (*n* = 679). **b**–**d** GSEA enrichment plots for male gastrocnemius muscle samples showing the KEGG Ca²⁺ signaling pathway (**b**); GO-BP striated muscle cell proliferation (**c**); and GO-BP muscle cell proliferation (**d**). **e**–**g** GSEA enrichment plots for female gastrocnemius muscle samples showing KEGG Ca²⁺ signaling pathway (**e**); GO-BP striated muscle cell proliferation (**f**); and GO-BP muscle cell proliferation (**g**). **h–j** Age- and sex-specific expression levels of the quiescence-associated regulator *SPRY1* (**h**); the early myogenic regulator *PAX3* (**i**); and the proliferative marker *CCND1* (**j**) in aged (old) muscle compared with young muscle. *P < 0.05, **P < 0.01, and ***P < 0.001 using two-tailed unpaired Student’s *t*-test. *TPM* transcripts per million.

To investigate the pathological consequences of Ca^2+^ dysregulation on myogenesis and its potential role in age-related muscle damage, we analyzed the human gastrocnemius transcriptome from the GTEx v8 dataset (*n* = 679). We categorized all GTEx skeletal muscle data by age and performed GSEA (Fig. 1a and Supplementary Table 2). In aged skeletal muscle, significant enrichment of the Ca^2+^ signaling pathway in the Kyoto Encyclopedia of Genes and Genomes (KEGG) pathway database, as well as of striated muscle cell proliferation and muscle cell proliferation terms in the Gene Ontology Biological Process (GO-BP) database in the aged group, was observed (Fig. 1b–g). Furthermore, significant upregulation of the quiescence-associated regulator *SPRY1*, the early myogenic regulator *PAX3*, and the proliferation marker *CCND1* was observed in aged muscle (Fig. 1h–j)^37,38^. Collectively, these profiles indicate an increased tendency for both self-renewal and activation of progenitor cells in aged skeletal muscle. These signals were evident in both male and female samples, suggesting that the transcriptional signature of Ca^2+^ signaling pathway activation and a proliferative bias is a general feature of aged muscle rather than a sex-restricted phenomenon.

**Fig. 2:**
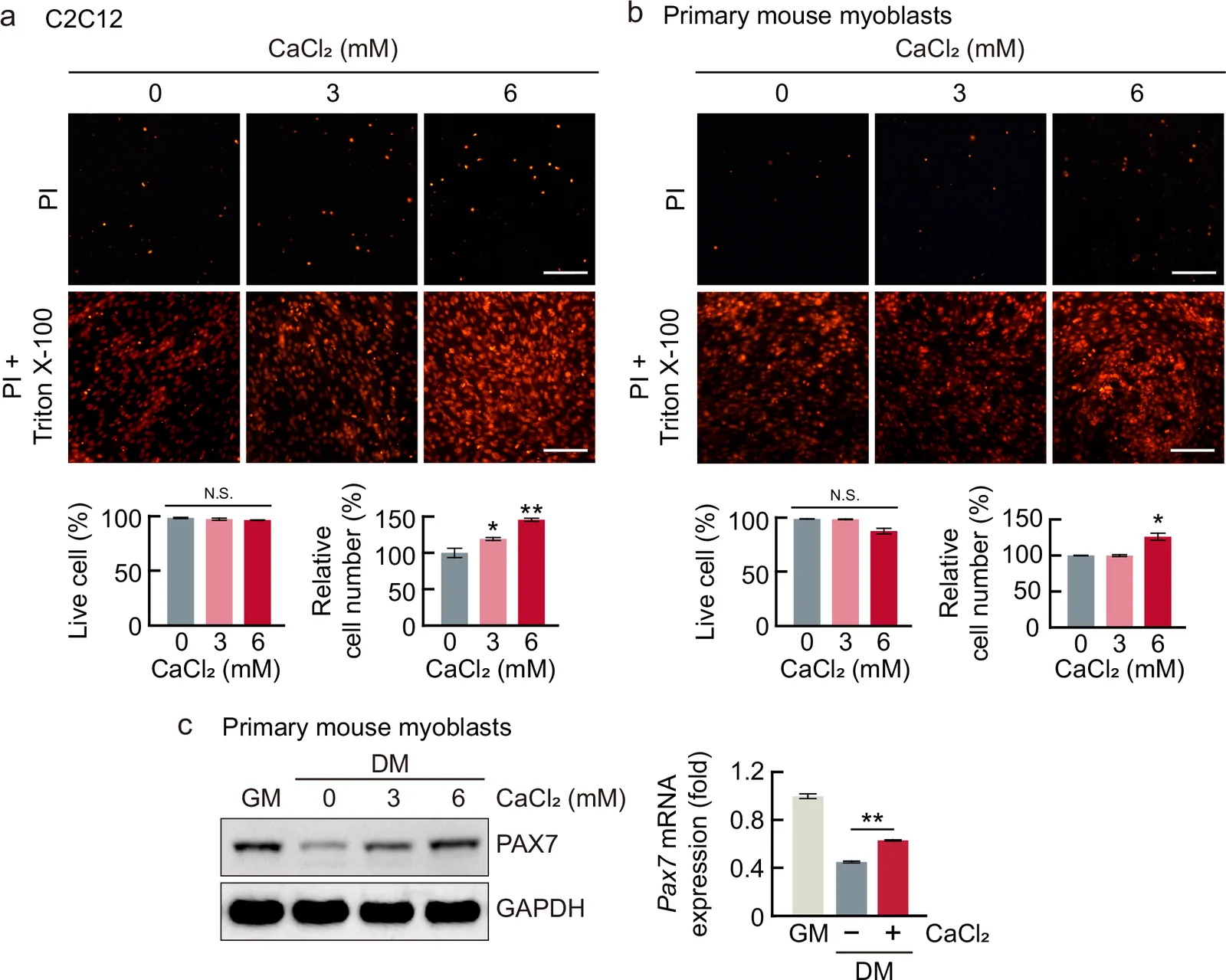
Excess Ca^2+^ exposure promotes proliferation and sustains a proliferative state of in vitro myoblasts. **a** PI staining of C2C12 cells after 5 days of differentiation with 0, 3, or 6 mM CaCl₂. **b** PI staining of primary mouse myoblasts differentiated for 3 days. Dead cells were detected by PI staining without fixation. Total nuclei were also quantified using PI staining after fixation and permeabilization. Scale bars = 200 μm. **c** Immunoblot analysis of the proliferative marker PAX7 protein levels (left) and relative *Pax7* mRNA expression levels (right) in primary mouse myoblasts. GM indicates growth medium (undifferentiated cells), and DM indicates differentiation medium (differentiated cells). Data are presented as mean ± s.e.m. *P < 0.05 and **P < 0.01 using one-way ANOVA followed by Tukey’s post hoc test. *NS* not significant.

Next, we investigated whether increased intracellular Ca²^+^ levels regulated cell proliferation. Under growth conditions, primary mouse myoblasts isolated from the hindlimb muscles and C2C12 myoblasts were exposed to a range of CaCl₂ concentrations. No significant increase in cell proliferation was observed in both C2C12 cells and primary myoblasts (Supplementary Fig. 1). We then exposed myoblasts to Ca^2+^ supplementation under differentiation conditions. After 5 days of differentiation under Ca^2+^ exposure, we assessed C2C12 cells using PI staining, quantifying the proportion of live cells and normalizing it to the total number of nuclei from parallel fixed and permeabilized cultures (Fig. 2a). Although no significant change was observed in the proportion of live cells, the total cell number was significantly increased in the presence of 3 and 6 mM CaCl₂. Similarly, PI staining of the primary mouse myoblasts after 3 days of differentiation under Ca^2+^ exposure demonstrated a significant increase in the total cell number without a significant change in the proportion of live cells (Fig. 2b and Supplementary Fig. 2a). Furthermore, despite the differentiation conditions, Ca^2+^ exposure significantly upregulated PAX7, a marker of proliferative myoblasts, at both the protein and mRNA levels in primary mouse myoblasts after 3 days of differentiation (Fig. 2c). These findings suggest that Ca^2+^ excess affects myoblast proliferation under differentiation conditions, not under growth conditions.

### Excess intracellular Ca^2+^ inhibits myogenic differentiation

**Fig. 3:**
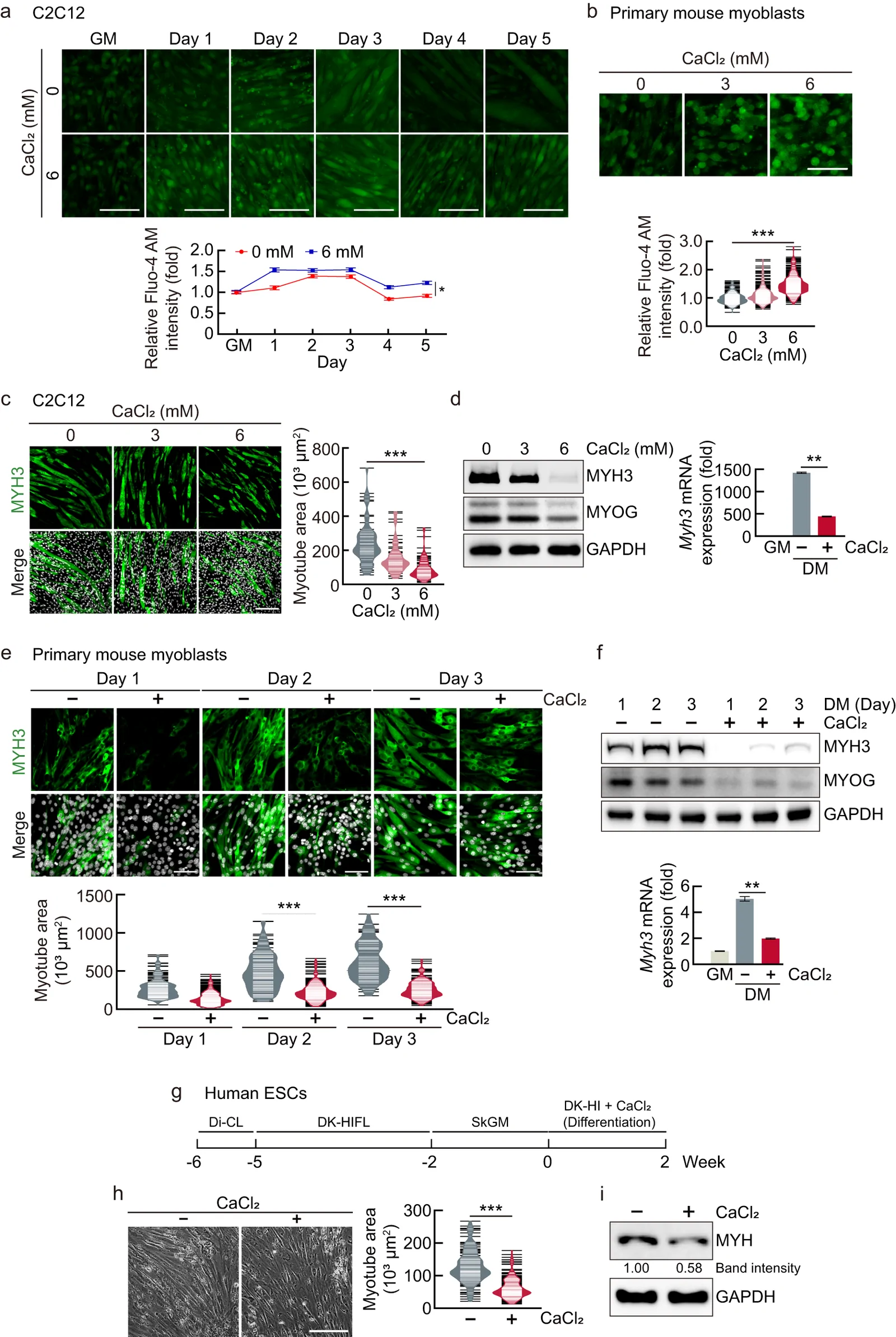
Excess Ca^2+^ exposure suppresses myogenesis in myoblasts. **a**, **b** Intracellular Ca²⁺ levels in myoblasts after Ca²⁺ exposure. **a** Time-course analysis of intracellular Ca²⁺ dynamics in C2C12 myoblasts during differentiation. C2C12 cells were differentiated for 5 days in the presence or absence of 6 mM CaCl₂ and labeled with the Fluo-4 AM Ca²⁺ indicator. Scale bars = 100 μm. Data are presented as mean ± s.e.m. and *P < 0.05 using two-tailed unpaired Student’s *t*-test. **b** Primary mouse myoblasts were differentiated for 3 days with 0, 3, or 6 mM CaCl₂ added to the differentiation medium and then labeled with the Fluo-4 AM Ca²⁺ indicator. Scale bars = 100 μm. Relative Fluo-4 AM fluorescence intensity was quantified with ImageJ software. **c** Myotube formation in C2C12 cells after Ca²⁺ exposure. C2C12 cells were differentiated for 5 days with 0, 3, or 6 mM CaCl₂ added to the differentiation medium and immunostained for MYH3 (green) and DAPI (white). Myotube area was quantified for each condition. **d** Immunoblot analysis of the myogenic marker proteins MYH3 and myogenin (MYOG, left) and relative *Myh3* mRNA expression levels (right) in C2C12 cells after Ca²⁺ exposure. **e** Myotube formation in primary mouse myoblasts after Ca²⁺ exposure. Primary mouse myoblasts were differentiated for 3 days with 0 or 6 mM CaCl₂ and immunostained for MYH3 (green) and DAPI (white). Myotube area was quantified for each condition. **f** Immunoblot analysis of MYH3 and MYOG protein levels (left) and relative *Myh3* mRNA expression levels (right) in primary mouse myoblasts differentiated for 3 days with Ca²⁺ exposure. **P < 0.01 and ***P < 0.001 using one-way ANOVA followed by Tukey’s post hoc test. **g** Schematic diagram of the myogenic differentiation protocol for hESCs, initiated at −6 weeks. hESCs were sequentially cultured in Di-CL medium (–6 to –5 weeks), DK-HIFL medium (−5 to −2 weeks), and SkGM (−2 to 0 weeks), followed by differentiation for 2 weeks in DK-HI medium supplemented with CaCl₂. **h** Representative images of myotubes in hESC-derived myoblasts after Ca²⁺ exposure (left) and quantification of myotube area (right). hESC-derived myoblasts were differentiated for 2 weeks with 0 or 3 mM CaCl₂. Data are presented as mean ± s.e.m. Scale bars = 200 μm. **i** Immunoblot analysis of MYH protein levels in hESC-derived myoblasts after Ca²⁺ exposure. Relative MYH expression was quantified with the ImageJ software. GAPDH served as a loading control. ***P < 0.001 using two-tailed unpaired Student’s *t*-test. *DM* differentiation medium (differentiated cells), *GM* growth medium (undifferentiated cells), *SkGM* skeletal muscle cell growth medium.

As myoblast proliferation was observed under differentiation conditions in response to excess Ca^2+^, we examined changes in the intracellular Ca^2+^ levels in myoblasts under myogenic differentiation conditions. Intracellular Ca^2+^ levels in C2C12 cells and primary mouse myoblasts were measured using fluorescence imaging with the Fluo-4 AM Ca^2+^ indicator. We monitored temporal changes in intracellular Ca^2+^ levels in C2C12 cells over 5 days of differentiation under conditions supplemented with 6 mM CaCl₂. Although the intracellular Ca^2+^ levels remained relatively stable in the control group, the Ca^2+^-exposed group consistently exhibited significantly higher Fluo-4 AM intensity at all time points from day 1 to day 5 (Fig. 3a). Similarly, in primary mouse myoblasts differentiated for 3 days under conditions supplemented with 3 or 6 mM CaCl₂, Fluo-4 AM fluorescence intensity increased by 1.12-fold and 1.48-fold, respectively (Fig. 3b). These data confirm that the intracellular Ca^2+^ levels in myoblasts significantly increased in a dose-dependent manner under differentiation conditions following Ca^2+^ exposure.

To investigate the effect of increased intracellular Ca^2+^ levels on myogenesis in vitro, we examined myogenic differentiation markers and myotube formation in response to Ca^2+^ supplementation. When C2C12 cells were cultured under differentiation conditions for 5 days with CaCl₂, a dose-dependent decrease in myotube formation was observed (Fig. 3c). The protein levels of the two myogenic markers MYH3 and myogenin(MYOG), as well as *Myh3* and *Myog* mRNA levels, were significantly reduced after CaCl₂ supplementation (Fig. 3d and Supplementary Fig. 2b). Similarly, primary mouse myoblasts showed impaired myotube formation and downregulation of myogenic markers when exposed to Ca^2+^ from days 1 to 3 (Fig. 3e, f). These results suggest that increased intracellular Ca^2+^ inhibits myogenesis in myoblasts.

To confirm Ca^2+^-induced suppression of myogenesis in human myoblasts, we generated hESC-derived myoblasts and induced myogenesis in the presence of 3 mM CaCl₂ for 2 weeks (Fig. 3g and Supplementary Fig. 2c). We observed impaired myotube formation in hESC-derived myoblasts exposed to Ca^2+^, with decreased total MYH protein levels (Fig. 3h, i). These findings confirm that Ca^2+^ exposure inhibits myogenesis in both mouse and human myoblasts.

### Excess intracellular Ca^2+^ activates the NFATc1 signaling pathway in mouse myoblasts

**Fig. 4:**
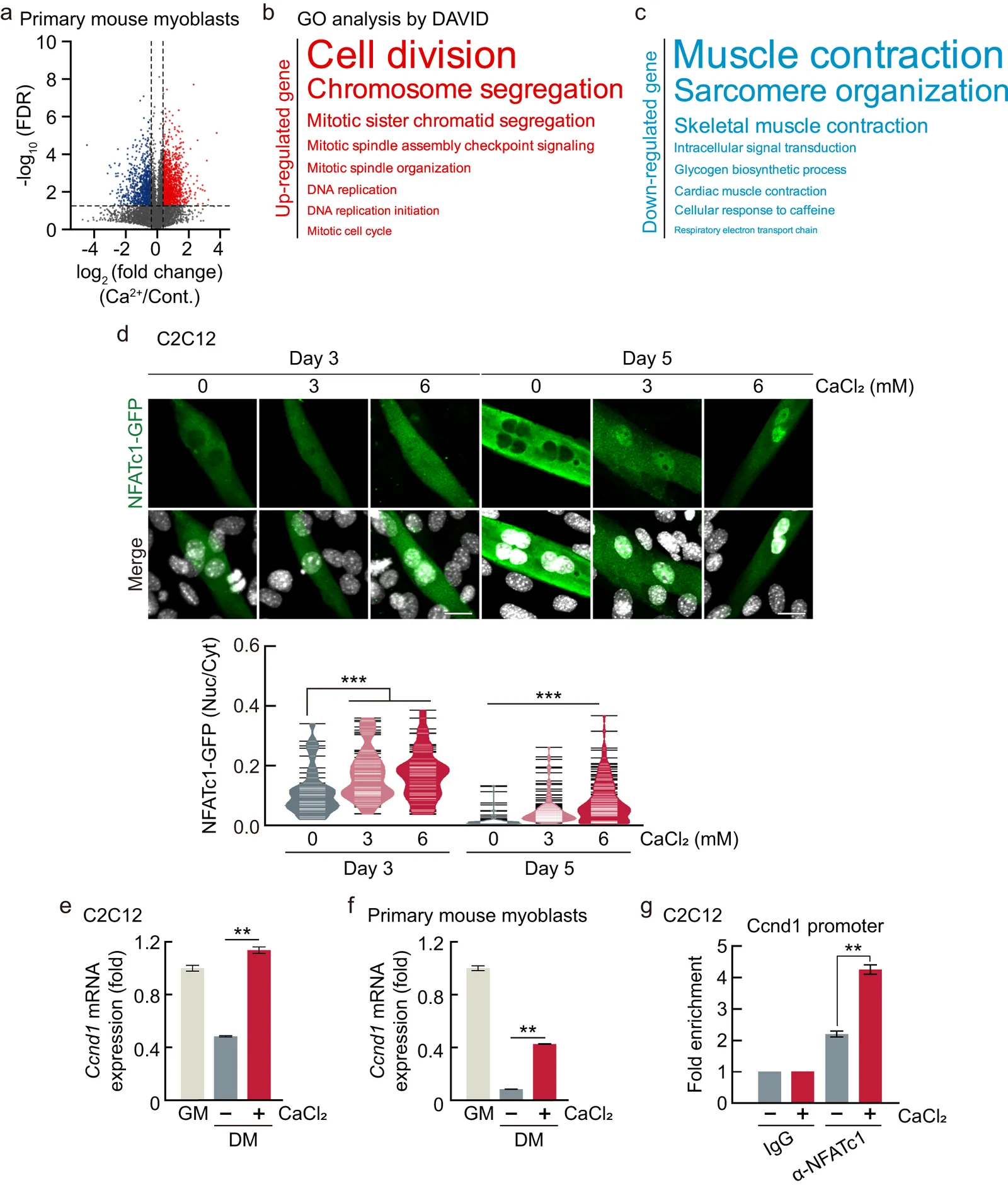
In vitro transcriptome profiling shows activation of proliferation pathways and the NFAT signaling pathway in Ca^2+^-exposed myoblasts. **a** Volcano plot showing DEGs in primary mouse myoblasts after Ca^2+^ exposure (FDR < 0.05, fold change > 1.35). Red and blue dots represent upregulated and downregulated genes, respectively. **b**, **c** Functional enrichment analysis of the top 500 genes upregulated (**b**) and downregulated (**c**) by Ca^2+^ exposure using DAVID. GO terms were sized proportionally to their significance (−log [FDR]). Primary mouse myoblasts were differentiated for 3 days with 0 or 6 mM CaCl₂. **d** Subcellular localization of NFATc1-GFP in C2C12 cells expressing NFATc1-GFP differentiated for 3–5 days with 0, 3, or 6 mM CaCl₂ and immunostained with GFP (green) and DAPI (white). The nuclear-to-cytoplasmic fluorescence intensity ratio was quantified with ImageJ software. Scale bars = 20 μm. **e**, **f** Relative mRNA expression of the NFAT target gene *Ccnd1* in C2C12 cells (**e**) and primary mouse myoblasts (**f**) differentiated for 5 and 3 days, respectively, with 0 and 6 mM CaCl₂. **g** ChIP analysis of NFATc1 recruitment to the Ccnd1 promoter. C2C12 cells were differentiated for 5 days with 0 or 6 mM CaCl₂, and chromatin was immunoprecipitated using control IgG or anti-NFATc1 antibody. PCR amplification of the Ccnd1 promoter region was performed on the immunoprecipitated chromatin. Data are presented as mean ± s.e.m. **P < 0.01 and ***P < 0.001 using one-way ANOVA followed by Tukey’s post hoc test. *DM* differentiation medium (differentiated cells), *FDR* false discovery rate, *GM* growth medium (undifferentiated cells).

To identify the mechanism of Ca^2+^-mediated myogenesis suppression, RNA sequencing (RNA-seq) was performed on primary mouse myoblasts differentiated for 3 days under Ca^2+^ exposure, and compared with control groups that were also differentiated for 3 days. We identified a total of 2,776 DEGs (Fig. 4a and Supplementary Table 3). Functional enrichment analysis using DAVID revealed that the top 500 genes upregulated by Ca^2+^ exposure were significantly enriched in proliferation-related terms, including cell division and chromosome segregation, whereas the top 500 downregulated genes were associated with myogenesis-related terms such as muscle contraction and sarcomere organization (Fig. 4b, c and Supplementary Table 4). Accordingly, GSEA supported this transcriptional shift, which showed consistent trends toward the upregulation of DNA replication, the suppression of muscle cell differentiation, and the activation of the Ca^2+^-dependent NFAT signaling pathway (Supplementary Fig. 3). These findings suggest that Ca^2+^ exposure activates the Ca^2+^–NFAT signaling pathway and sustains a proliferative state in primary mouse myoblasts during myogenesis.

Next, to verify in vitro the activation of the Ca^2+^-dependent NFAT signaling pathway in myoblasts after Ca^2+^ exposure, we first assessed the nuclear translocation of the Ca^2+^-dependent transcription factor NFATc1 in C2C12 cells. C2C12 cells expressing NFATc1-GFP were differentiated for 3 and 5 days with 3 or 6 mM CaCl₂, and nuclear translocation of NFATc1-GFP was observed (Fig. 4d). We also observed nuclear translocation of endogenous NFATc1 in C2C12 cells on Ca^2+^ exposure (Supplementary Fig. 4). These results indicate that Ca^2+^ exposure activates the NFAT signaling pathway in myoblasts. Furthermore, expression of the NFAT signaling pathway target gene *Ccnd1* was significantly upregulated in both C2C12 cells and primary mouse myoblasts differentiated under Ca^2+^ exposure (Fig. 4e, f). To assess direct transcriptional regulation, we performed ChIP analysis using an anti-NFATc1 antibody in C2C12 cells differentiated under Ca^2+^ exposure (Fig. 4g). NFATc1 binding to the Ccnd1 promoter was significantly enhanced under Ca^2+^ exposure compared with control conditions. Collectively, these results confirm that Ca^2+^ exposure induces activation of the NFATc1 signaling pathway in mouse myoblasts, thereby upregulating the expression of proliferation-associated genes.

### Pathological Ca^2+^ excess disrupts myogenic commitment of skeletal muscle tissue

**Fig. 5:**
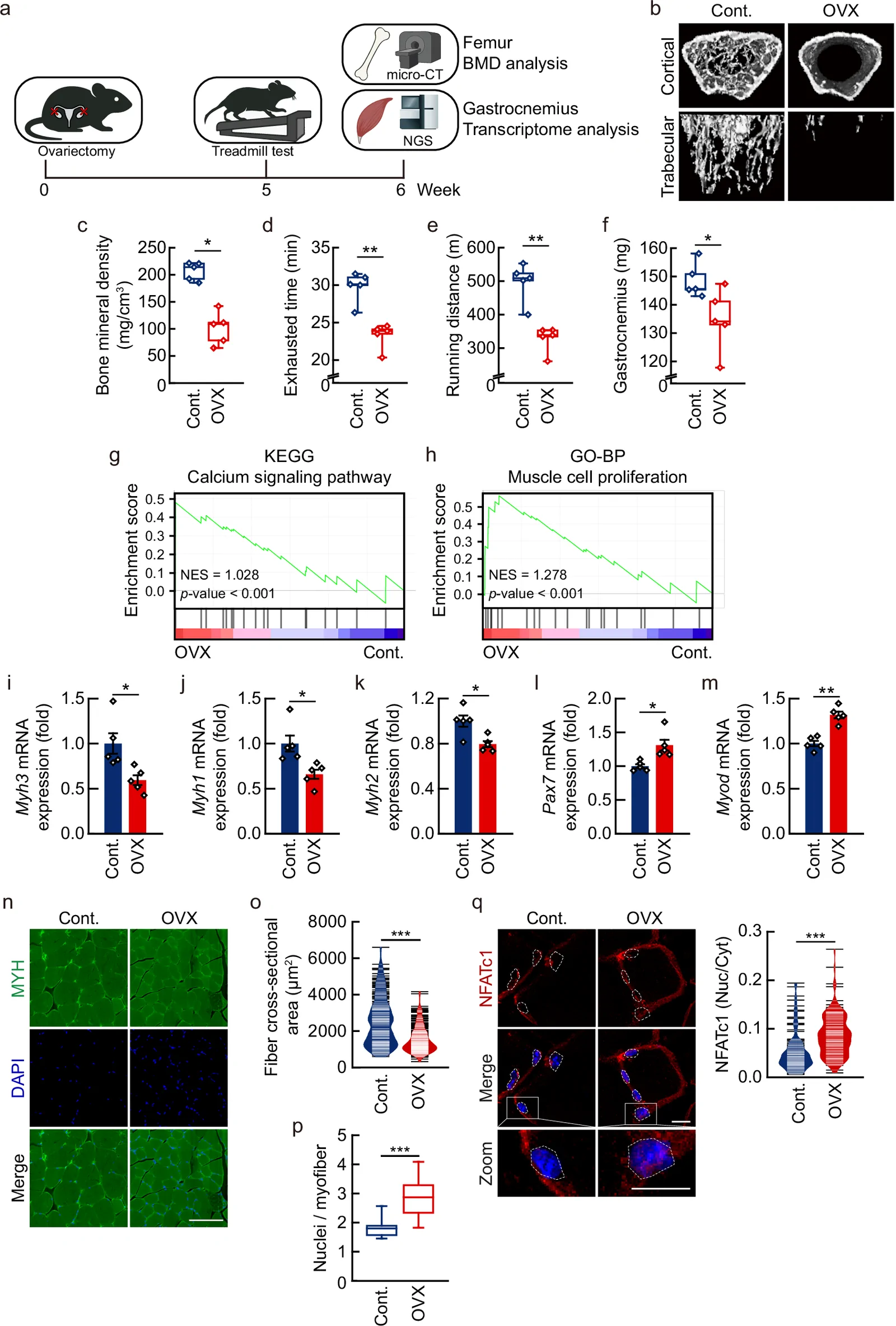
Ovariectomized mice exhibit muscle atrophy with activated Ca^2+^–NFAT signaling. **a** Schematic of the experimental procedure. **b** Representative micro-CT images of cortical and trabecular regions of the femur (*n* = 5 per group). **c** Bone mineral density (BMD) of the femur in OVX and control mice. **d–f** Muscle endurance assessment using treadmill testing: time to exhaustion (**d**), total running distance (**e**), and gastrocnemius muscle weight (**f**). **g**, **h** GSEA enrichment plots of DEGs for the KEGG Ca^2+^ signaling pathway (**g**) and the GO-BP muscle cell proliferation (**h**). **i–m** mRNA expression of myogenic and proliferative markers in OVX mice. Expression of myogenic markers *Myh3* (**i**), *Myh1* (**j**), and *Myh2* (**k**) and proliferative markers *Pax7* (**l**) and *MyoD* (**m**) in the gastrocnemius muscle. The 18S rRNA was used as the reference gene for qRT-PCR. **n–p** Histological analysis of the gastrocnemius muscles from OVX mice. Cross-sections were immunostained with MYH3 (green) and DAPI (blue) (**n**); quantification of myofiber cross-sectional area (**o**) and the number of nuclei per myofiber (**p**). Scale bars = 100 μm. Boxes represent the median with 25th and 75th percentiles (quartiles) and the minimum and maximum values. **q** Representative immunofluorescence images of gastrocnemius muscle cross-sections immunostained for NFATc1 (red) and DAPI (blue). Nuclear translocation of NFATc1 protein was quantified using the nuclear-to-cytoplasmic fluorescence intensity ratio. Outlines indicate satellite cell nuclei. Scale bars = 10 μm. The myofiber cross-sectional area and nuclear-to-cytoplasmic fluorescence intensity ratio were quantified with ImageJ software. Data are presented as mean ± s.e.m. *P < 0.05, **P < 0.01, and ***P < 0.001 using a two-tailed unpaired Student’s *t*-test.

Estrogen deficiency through OVX increases sarcoplasmic reticulum (SR) Ca^2+^ stores and promotes spontaneous Ca^2+^ release from SR^39,40^. Specifically, in the skeletal muscle, OVX disturbs Ca^2+^ homeostasis by increasing the Ser2844 phosphorylation of the SR Ca^2+^ release channel ryanodine receptor 1 (RyR1)^41^. Therefore, we established an OVX mouse model to investigate muscle changes in response to the dysregulation of Ca^2+^ metabolism (Fig. 5a). We observed significant bone loss, indicated by decreased bone mineral density thereby confirming the successful OVX (Fig. 5b, c). OVX induced significant increases in body weight and white adipose tissue mass, whereas the heart, liver, and spleen weights remained unchanged and all serum parameters in both groups remained within the normal range (Supplementary Fig. 5). However, OVX mice showed reduced muscle performance in the treadmill test and decreased gastrocnemius muscle weight, indicating that muscle atrophy was induced (Fig. 5d–f).

Next, we analyzed the gastrocnemius muscles of OVX mice to investigate the mechanism inducing muscle atrophy in vivo. By analyzing the gastrocnemius transcriptome of OVX mice, a total of 1,985 DEGs were identified, and GSEA analysis was used to evaluate the enrichment of gene sets from the GO-BP and KEGG pathway databases (Supplementary Fig. 6 and Supplementary Tables 5 and 6). GSEA analysis revealed positive enrichment of gene sets related to the Ca^2+^ signaling pathway (KEGG database) and muscle cell proliferation (GO-BP database) in OVX mice (Fig. 5g, h). We observed a significant downregulation of mRNA expression of the developing myotube marker *Myh3* and the mature myotube markers *Myh1* and *Myh2*, along with significant upregulation of mRNA expression for the proliferative markers *Pax7* and *MyoD* in OVX muscle tissue (Fig. 5i–m). Histological analysis of muscle sections revealed significant myofiber atrophy in OVX mice with a reduced myofiber cross-sectional area (Fig. 5n, o). In addition, the number of nuclei per myofiber was significantly increased in the skeletal muscle of OVX mice (Fig. 5p). We also observed significant nuclear translocation of NFATc1 in the muscle tissue of OVX mice, indicating activated NFAT pathway (Fig. 5q). These results were consistent with in vitro Ca^2+^ supplementation experiments in myoblasts, suggesting that abnormal activation of the NFAT signaling pathway in the skeletal muscle of OVX mice contributes to the persistent proliferative state and suppressed myogenesis.

**Fig. 6:**
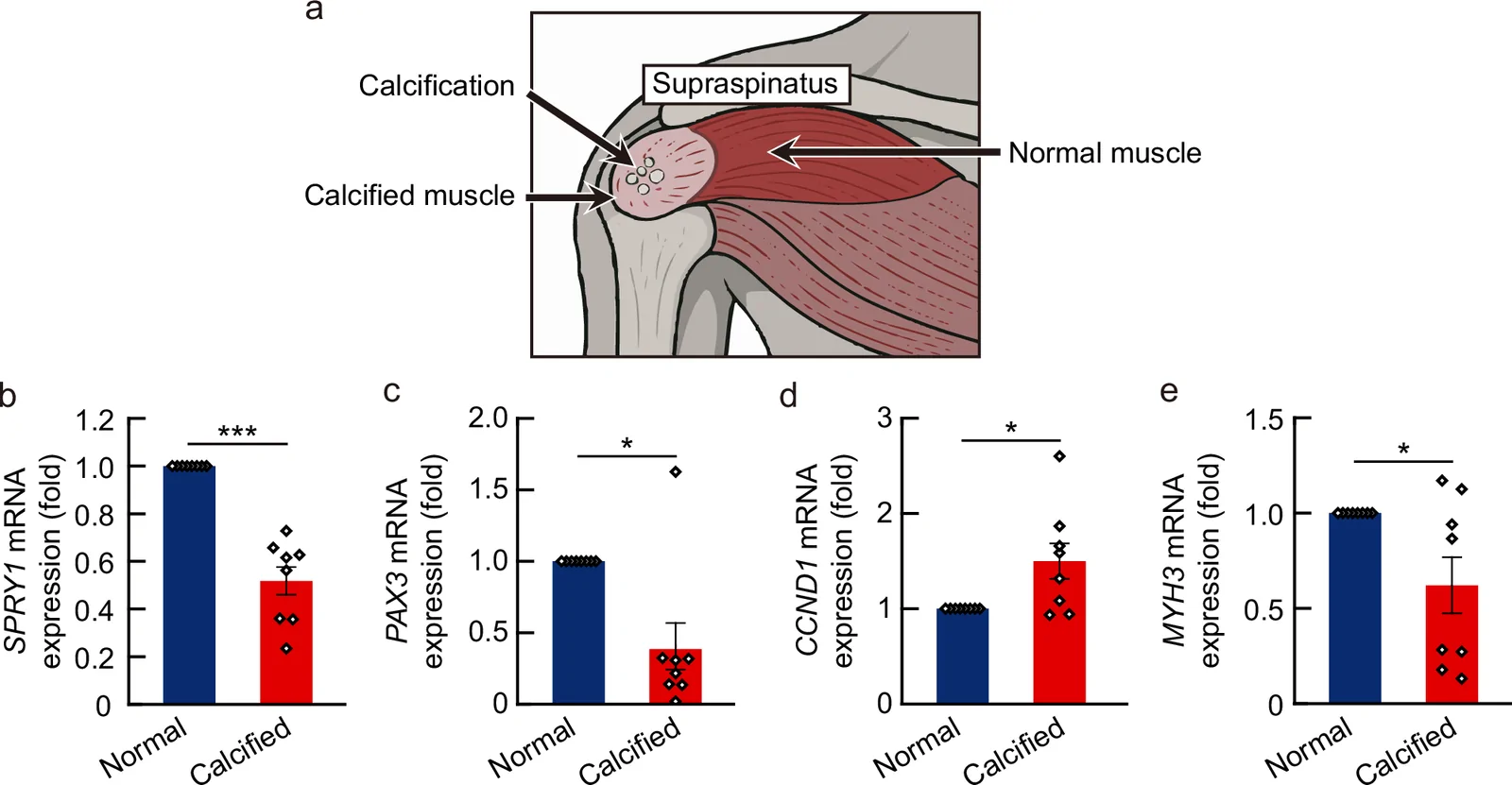
Calcific tendinitis induces proliferation while inhibiting myogenesis in adjacent skeletal muscles. **a** Schematic representation of calcification in the supraspinatus muscle from patients with calcific tendinitis. **b**–**e** mRNA expression levels in calcified muscles from patients with calcific tendinitis (*n* = 8). Expression of quiescent markers *SPRY1* (**b**) and *PAX3* (**c**), proliferative marker *CCND1* (**d**), and myogenic marker *MYH3* (**e**) in calcified muscle compared with those in normal muscle. The 18S rRNA was used as the reference gene for qRT-PCR. Data are presented as mean ± s.e.m. *P < 0.05 and ***P < 0.001 using a two-tailed unpaired Student’s *t*-test.

Because the contribution of factors other than Ca^2+^ dysregulation to impaired myogenesis cannot be excluded in the OVX mouse model, we further investigated human in vivo conditions characterized by localized Ca^2+^ overload. Calcific tendinitis is characterized by repeated mineral resorption and re-crystallization, which leads to localized increases in Ca^2+^ levels within adjacent muscles^42^. We compared the calcified areas of the supraspinatus muscle from patients with calcific tendinitis with adjacent noncalcified controls to assess the impact of pathological Ca^2+^ overload by analyzing mRNA expression patterns in the calcified muscle (Fig. 6a). Calcified muscle samples exhibited downregulation of the quiescence-associated regulator *SPRY1* and the early myogenic regulator *PAX3*, and upregulation of the proliferative marker *CCND1* with downregulation of the myogenic marker *MYH3* (Fig. 6b–e). These findings suggest that pathological Ca^2+^ deposition contributes to impaired myogenic maintenance by activating proliferative pathways in progenitor cells of the skeletal muscle.

### NFAT inhibitor VIVIT alleviates Ca^2+^-induced inhibition of myogenesis in vitro and in vivo

**Fig. 7:**
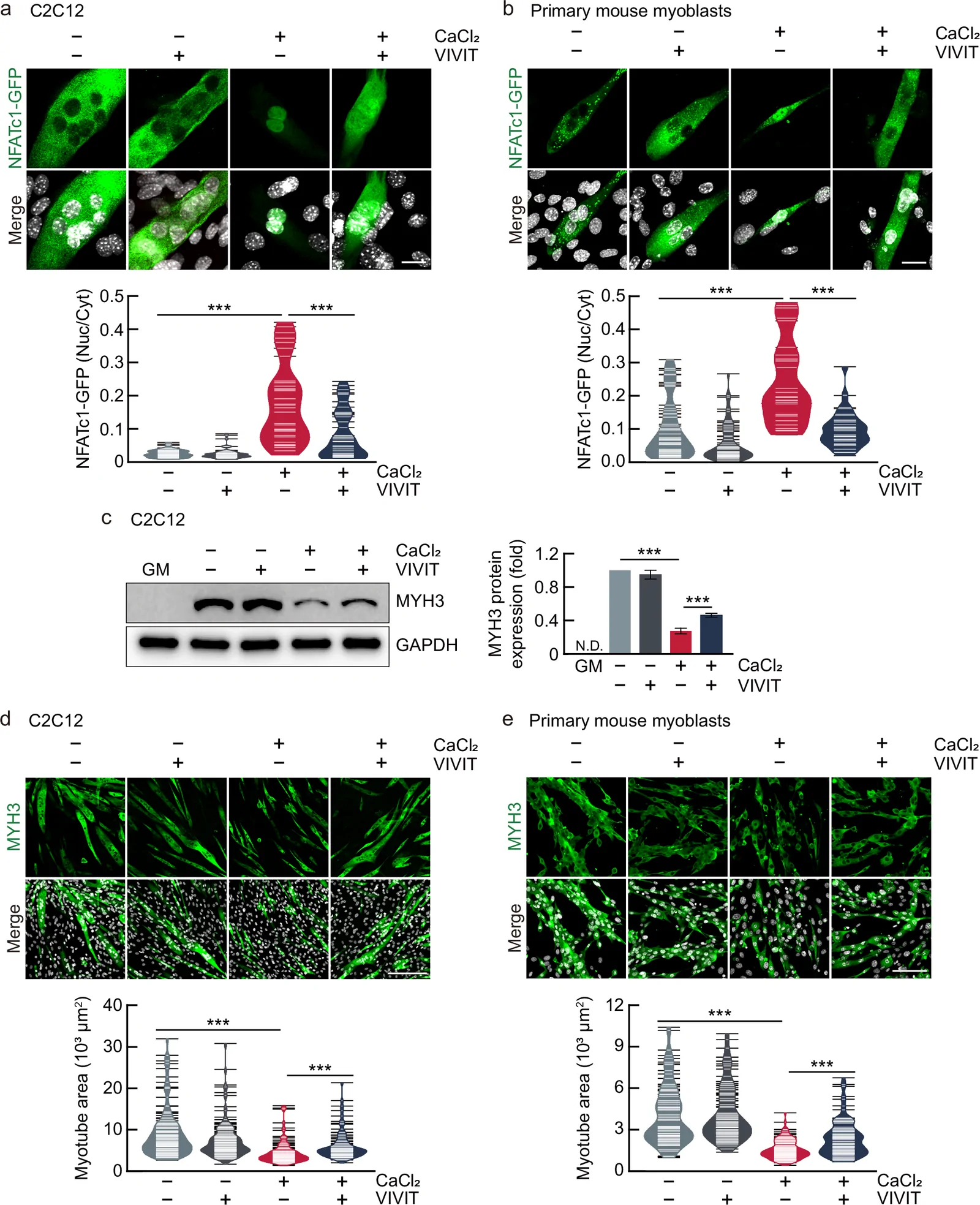
NFAT pathway inhibition by VIVIT restores myogenesis in Ca^2+^-exposed myoblasts in vitro. **a** **b** VIVIT-mediated inhibition of NFAT nuclear translocation. C2C12 cells (**a**) and primary mouse myoblasts (**b**) expressing NFATc1-GFP were differentiated with 6 mM CaCl₂ and 20 μM VIVIT, an NFAT inhibitor, and immunostained with GFP (green) and DAPI (white). The nuclear-to-cytoplasmic fluorescence intensity ratio was quantified. Scale bars = 20 μm. **c** Immunoblot analysis of MYH3 in C2C12 cells differentiated under indicated conditions. GAPDH served as a loading control. Relative MYH3 expression was quantified. Data are presented as mean ± s.e.m. **d**, **e** VIVIT-mediated restoration of myogenesis. C2C12 cells (**d**) and primary mouse myoblasts (**e**) were differentiated under indicated conditions and immunostained with MYH3 (green) and DAPI (white). Myotube area was quantified. Scale bars = 200 μm. C2C12 cells and primary mouse myoblasts were differentiated for 5 and 3 days, respectively. Images were measured with ImageJ software. ***P < 0.001 using one-way ANOVA followed by Tukey’s post hoc test.

As Ca^2+^ excess inhibits myogenesis and promotes proliferation via the NFAT signaling pathway, we investigated the potential of NFAT as a therapeutic target for muscle weakness using the selective NFAT inhibitor VIVIT peptide^43^. C2C12 cells and primary mouse myoblasts were differentiated in the presence of CaCl₂ supplementation and 20 µM VIVIT. To verify on-target NFAT pathway inhibition, we quantified NFATc1 subcellular localization in cells expressing NFATc1-GFP. VIVIT treatment effectively inhibited Ca^2+^-induced NFATc1 nuclear translocation in both C2C12 cells and primary mouse myoblasts expressing NFATc1-GFP, showing significant inhibition of the NFAT signaling pathway (Fig. 7a, b). VIVIT restored Ca^2+^-induced suppression of the early myofiber marker MYH3, demonstrating recovery of the myogenic program under Ca^2+^ overload (Fig. 7c). Finally, we assessed morphological differentiation. VIVIT reversed the defect in myotube formation observed under Ca^2+^ exposure (Fig. 7d, e). To validate this rescue effect using a physiologically relevant model, we used caffeine-induced SR Ca^2+^ leak^44,45^. Caffeine treatment (1 mM) markedly suppressed myoblast fusion and MYH3 expression—defects that were successfully rescued by VIVIT (Supplementary Fig. 7).

**Fig. 8:**
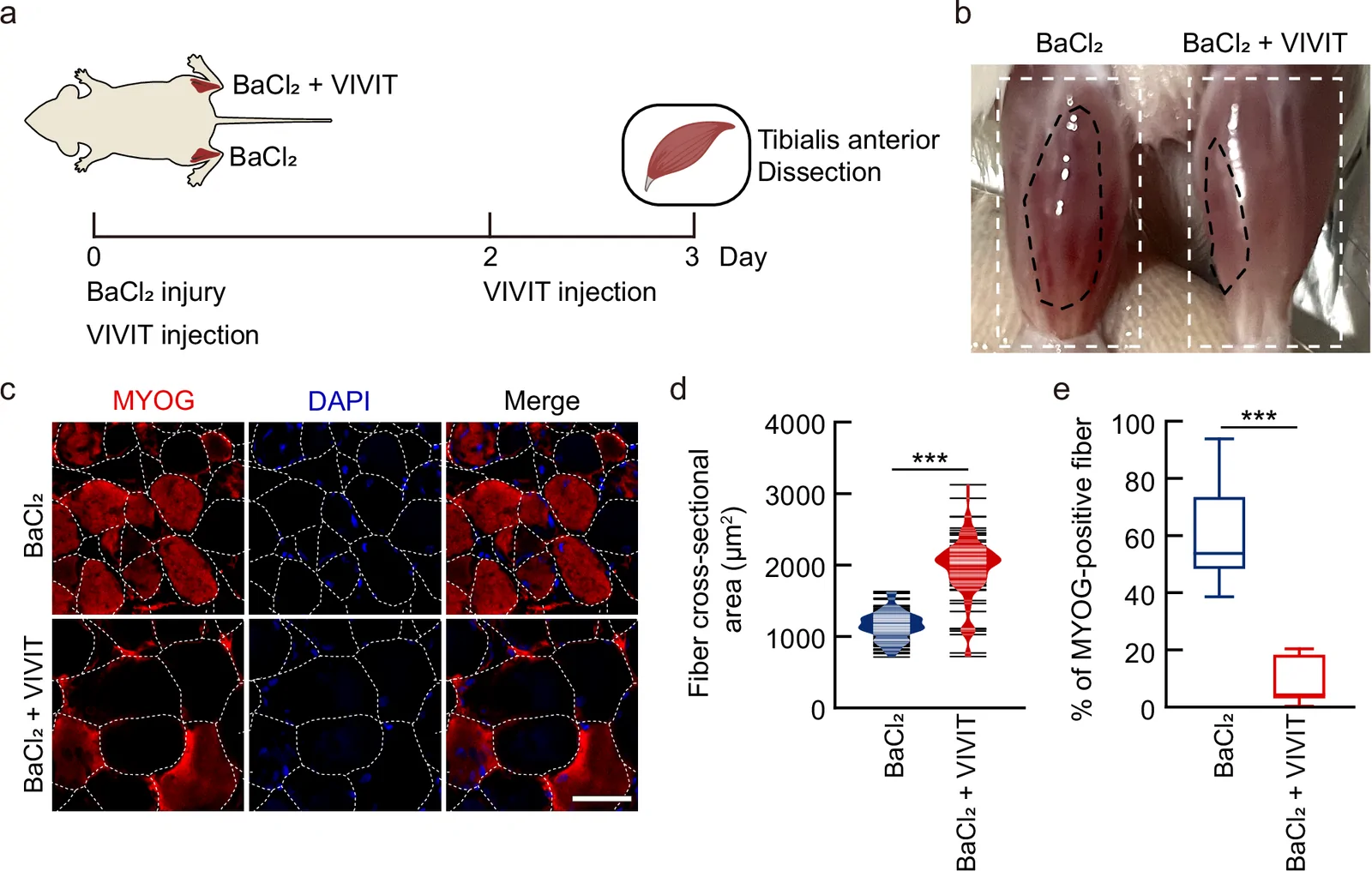
VIVIT promotes myofiber maturation in the BaCl₂-induced muscle injury mouse model. **a** Schematic of the experimental timeline for BaCl₂-induced muscle injury and VIVIT administration. The tibialis anterior (TA) muscles were injured by 1.2% BaCl₂ injection, followed by local administration of VIVIT peptide (170 μg) or saline control intramuscularly on day 0 and day 2 post-injury. **b** Representative gross morphology of regenerating TA muscles at 3 days post-injury. **c** Representative immunofluorescence images of muscle sections stained for MYOG (red) and DAPI (blue). White dashed lines indicate regenerating myofiber outlines. Scale bars = 100 μm. **d**, **e** Quantitative analysis of myofiber maturation in regenerating TA muscles: myofiber cross-sectional area (**d**) and percentage of MYOG-positive fibers (**e**). Data were quantified with ImageJ software. Data are presented as mean ± s.e.m. ***P < 0.001 using a two-tailed unpaired Student’s *t*-test.

To validate the physiological relevance of our in vitro findings, we used an in vivo model of BaCl₂-induced acute skeletal muscle injury, which causes extensive myofiber necrosis and initiates a robust regenerative response^13^. To determine whether Ca^2+^-dependent signaling is activated in this injury model, we assessed calpain-mediated proteolysis by examining the cleavage of NF2, a known calpain substrate^46^. Immunoblot analysis revealed increased accumulation of the cleaved NF2 fragment in injured muscles compared with contralateral uninjured controls, consistent with the activation of Ca^2+^-dependent proteolytic signaling after BaCl₂ injury (Supplementary Fig. 8a). We injected BaCl₂ into the TA muscles of mice, followed by local administration of either saline or VIVIT on days 0 and 2 post-injury (Fig. 8a). Consequently, VIVIT-treated TA muscles exhibited improved regenerative morphology compared with BaCl₂-only controls at day 3 (Fig. 8b). Because inflammatory responses contribute critically to skeletal muscle regeneration, we next examined whether the observed effects of VIVIT were associated with altered inflammatory signaling. Quantitative PCR analysis demonstrated that VIVIT treatment did not significantly affect the expression of the pro-inflammatory markers *Il6*, *Tnfa*, and *Inos* compared with the BaCl₂-only group (Supplementary Fig. 8b–d). To evaluate muscle maturation dynamics, we performed immunofluorescence analysis of MYOG, a marker whose downregulation is required for terminal myofiber maturation. VIVIT-treated muscles showed significantly increased myofiber cross-sectional area compared with BaCl₂-only controls (Fig. 8c,d). Furthermore, although BaCl₂-injured muscles exhibited a high proportion of MYOG-positive immature fibers, VIVIT administration dramatically reduced the proportion of MYOG-positive fibers (Fig. 8e). These results demonstrate that pharmacological NFAT inhibition via VIVIT promotes muscle regeneration after injury. Collectively, our in vivo data confirm that the Ca^2+^–NFAT axis is a critical mediator of impaired muscle regeneration under conditions of Ca^2+^ dysregulation and represents a therapeutic target to enhance regenerative capacity.

## Discussion

In this study, we demonstrate that sustained intracellular Ca^2+^ elevation disrupts skeletal muscle regeneration by maintaining the activation of the Ca^2+^–NFAT signaling axis, thereby retaining myoblasts in a proliferative state and preventing myogenic differentiation^47–49^. The Ca^2+^-dependent phosphatase calcineurin acts as a central mediator of this pathway, where prolonged Ca^2+^ elevation induces NFATc1 dephosphorylation and nuclear translocation, ultimately blocking cell cycle exit required for myogenesis. Consistent with this mechanism, transcriptomic analyses revealed enrichment of DNA replication and cell cycle-associated pathways alongside suppression of muscle differentiation programs, with *CCND1* emerging as a key NFAT target gene that shifts cellular resources toward proliferation. Although the calcineurin–NFAT pathway is classically recognized as a positive regulator of myogenesis, our findings reveal a duration-dependent dual function: transient activation supports differentiation, whereas sustained activation under pathological Ca^2+^ conditions inhibits myogenic progression.

Importantly, our study addresses a critical gap in understanding both the downstream effects of Ca^2+^ signaling and the pathological contexts in which Ca^2+^ homeostasis becomes dysregulated. Multiple independent lines of evidence demonstrate that diverse conditions, including aging, hormonal deficiency, and localized tissue pathology, converge on chronic intracellular Ca^2+^ elevation. In aged human skeletal muscle (GTEx dataset), we observed enrichment of Ca^2+^ signaling pathways consistent with prior reports of increased RyR1 leakiness and reduced SERCA-mediated Ca^2+^ reuptake efficiency. In the OVX model, estrogen deficiency destabilizes Ca^2+^ handling through RyR hyperactivation, resulting in spontaneous Ca^2+^ release into the cytosol. In calcific tendinitis, the dynamic resorptive phase generates localized increases in extracellular Ca^2+^ concentration that drive sustained Ca^2+^ influx into adjacent muscle cells. These contexts collectively support the concept that chronic Ca^2+^ dysregulation represents a biologically grounded and clinically relevant upstream driver of aberrant signaling.

To further ensure physiological relevance, we examined the effects of Ca^2+^ across multiple experimental systems with varying exposure durations and concentrations. Although short-term in vitro exposure required relatively higher CaCl₂ concentrations, prolonged exposure to lower concentrations in hESC-derived myogenic cultures was sufficient to impair differentiation. These findings indicate that chronic exposure duration, rather than absolute Ca^2+^ concentration, is a critical determinant of pathological outcomes. Baseline intracellular Ca^2+^ dynamics during normal differentiation were tightly regulated, confirming that sustained Ca^2+^ elevation represents a pathological deviation. Complementary experiments using caffeine-induced SR Ca^2+^ leak recapitulated the observed phenotypes, demonstrating that the effects result from intracellular Ca^2+^ dysregulation rather than nonspecific extracellular stress.

Remarkably, consistent molecular signatures across calcific tissues, aged human datasets, and experimental models demonstrate convergence on a common Ca^2+^–NFAT–proliferation axis. To establish causality at the organismal level, we performed in vivo validation using a BaCl₂-induced injury model of pathological Ca^2+^ influx. Pharmacological NFAT inhibition with VIVIT restored myofiber maturation, increased cross-sectional area, and reduced the proportion of immature MYOG-positive fibers. These findings extend our in vitro observations and establish that sustained NFAT activation functionally drives defective muscle regeneration under conditions of Ca^2+^ dysregulation.

These results have broader implications for understanding the interplay between muscle and bone pathophysiology^50,51^. The co-occurrence of osteoporosis and sarcopenia, often termed osteosarcopenia, remains mechanistically unresolved. Given that Ca^2+^ handling abnormalities contribute to bone loss, our findings suggest that similar dysregulation also impairs muscle regeneration through sustained NFAT activation, providing a unifying framework linking these conditions. However, this connection remains inferential and warrants further direct investigation. Although we successfully demonstrated VIVIT-mediated rescue in the BaCl₂-induced injury model, we were unable to evaluate this therapeutic effect in the chronic OVX model because of methodological limitations associated with long-term NFAT inhibition studies. Because VIVIT was administered locally by intramuscular injection, sustained NFAT inhibition throughout the 6-week OVX protocol would require repeated administrations over an extended period, potentially introducing local tissue injury, inflammation, and remodeling that could confound interpretation of muscle phenotypes. Future studies using long-term pharmacological approaches or genetic models will be valuable for directly assessing the role of NFAT signaling in OVX-associated muscle dysfunction.

In conclusion, our study identifies sustained activation of the Ca^2+^–NFAT pathway as a key mechanism by which chronic Ca^2+^ dysregulation impairs myogenic differentiation and muscle regeneration. By distinguishing between physiological and pathological Ca^2+^ signaling, we propose a duration-dependent model in which NFAT functions as a dual regulator of myogenesis. Targeting this pathway, as demonstrated with NFAT inhibition, represents a promising therapeutic strategy for conditions characterized by aberrant Ca^2+^ homeostasis. Future studies should define upstream regulatory mechanisms in greater detail, validate long-term in vivo efficacy, and explore translational applications in age-related sarcopenia and Ca^2+^-associated myopathies.

## Supplementary information

Supplementary Information

Supplementary Table 1

Supplementary Table 2

Supplementary Table 3

Supplementary Table 4

Supplementary Table 5

Supplementary Table 6

## Data Availability

The data generated in this study are available in the Article and its Supplementary Data. All raw RNA sequencing data have been deposited in the NCBI SRA database with accession numbers SRX30336155, SRX30336156, SRX30336157, SRX30336158, SRX30336159, SRX30336160, SRX30336161, and SRX30336162 under BioProject PRJNA1314403 (https://www.ncbi.nlm.nih.gov/bioproject/PRJNA1314403).
