# Supplementary material for: Pathological calcium overload impairs skeletal muscle regeneration through sustained NFAT-dependent proliferative signaling
Source: Exp Mol Med. 2026 Oct 2;58(10):3409–22. doi: 10.1038/s12276-026-01856-3 (PMC13737287; doi:10.1038/s12276-026-01856-3)
Supplement: Supplementary file 1 [file 12276_2026_1856_MOESM1_ESM.pdf]

**SUPPLEMENTARY INFORMATION**

# Pathological calcium overload impairs skeletal muscle regeneration through sustained NFAT-dependent proliferative signaling

Sangsoo Lee[1], Namjoon Cho[1], Seokwoo Jo[2], Jong-Hun Ji[3], Jong Ok Kim[4], Chungoo Park[2], Sunkyung Choi[5*], Eun-Mi Kim[6*], Kee K. Kim[1*]

[1]Department of Biochemistry, College of Natural Sciences, Chungnam National University, Daejeon, 34134, Republic of Korea

[2]School of Biological Science and Technology, Chonnam National University, Gwangju, Republic of Korea

[3]Department of Orthopedic Surgery, Daejeon St. Mary's Hospital, The Catholic University of Korea, Daejeon, Republic of Korea

[4]Department of Pathology, Daejeon St. Mary's Hospital, The Catholic University of Korea, Daejeon, Republic of Korea

[5]Department of Biological Sciences, College of Natural Sciences, Keimyung University, Daegu 42601, Republic of Korea

[6]Department of Bio and Environmental Technology, Seoul Women's University, Seoul, 01797, Republic of Korea

***Corresponding authors**. Email: kimkk@cnu.ac.kr (Kee K. Kim), eunmi.kim@swu.ac.kr (Eun-Mi Kim), and skchoi@kmu.ac.kr (Sunkyung Choi)

This document includes:

- Supplementary method
- Supplementary Figures 1 to 8
- Supplementary Tables list

## SUPPLEMENTARY METHODS

### Cell viability assays

Myoblast viability after $Ca^{2+}$ exposure was assessed using the MTS assay. C2C12 cells were cultured in $Ca^{2+}$-supplemented growth medium for 24 h, and the CellTiter 96® AQueous One Solution Cell Proliferation Assay kit (Promega, USA) was used according to the manufacturer's instructions. Absorbance at 490 nm was recorded using an EMax Plus Microplate Reader.

### Serum biochemical analysis

Serum biochemical parameters were analyzed using an autoanalyzer (AU680; Olympus, USA). The measured parameters included aspartate aminotransferase (AST), glucose, blood urea nitrogen (BUN), total cholesterol, triglycerides (TG), high-density lipoprotein cholesterol (HDL-C), low-density lipoprotein cholesterol (LDL-C) levels.

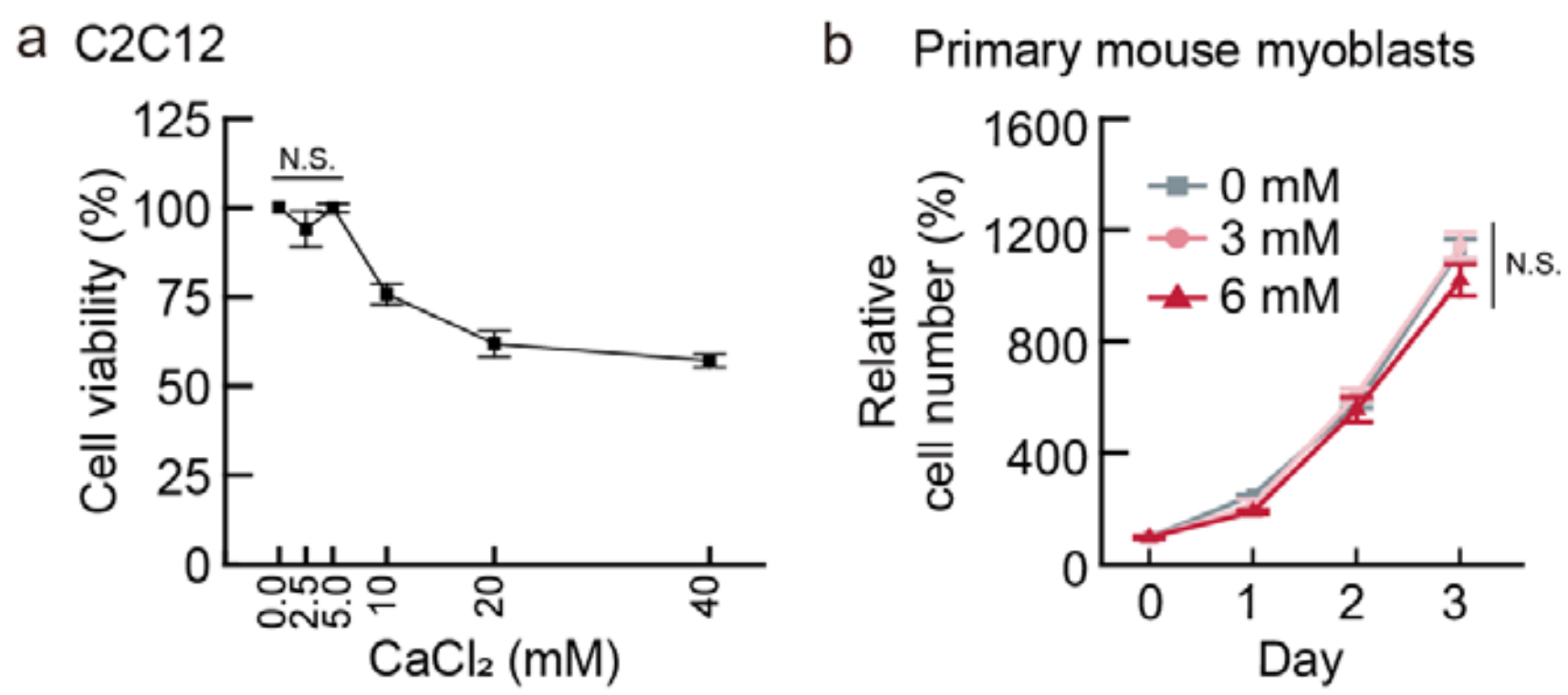


**Supplementary Fig. 1. $Ca^{2+}$ exposure does not affect cell proliferation of *in vitro* myoblasts under growth condition.**

**a** Cell viability of C2C12 cells assessed by MTS assay after 24 h exposure to 0–40 mM $CaCl_2$ added to the growth medium. **b** Growth curves of primary mouse myoblasts cultured with 0, 3, or 6 mM $CaCl_2$. Data are presented as mean ± SEM. N.S., not significant using one-way ANOVA followed by Tukey's post hoc test.

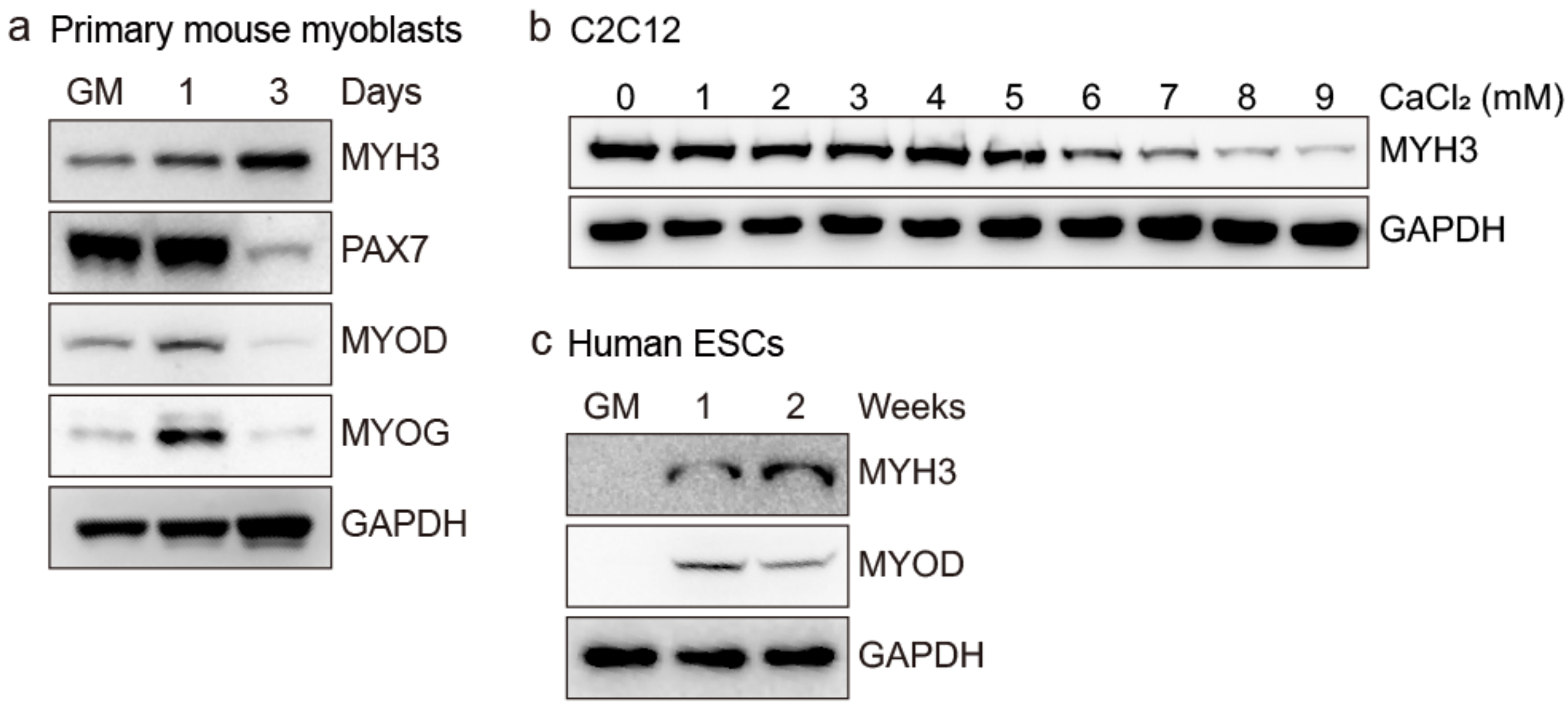


**Supplementary Fig. 2. Validation of myoblast myogenesis.**

**a** Immunoblot analysis of myogenic marker proteins MYH3 and PAX7 and MYOD, and MYOG in primary mouse myoblast differentiated for 1 and 3 days. **b** Immunoblot analysis MYH3 in C2C12 cells differentiated for 5 days with 0–9 mM $CaCl_2$. **c** Immunoblot analysis of MYH3 and MYOD in hESC-derived myoblasts differentiated for 1 and 2 weeks. GM, growth medium (undifferentiated cells). GAPDH served as a loading control.

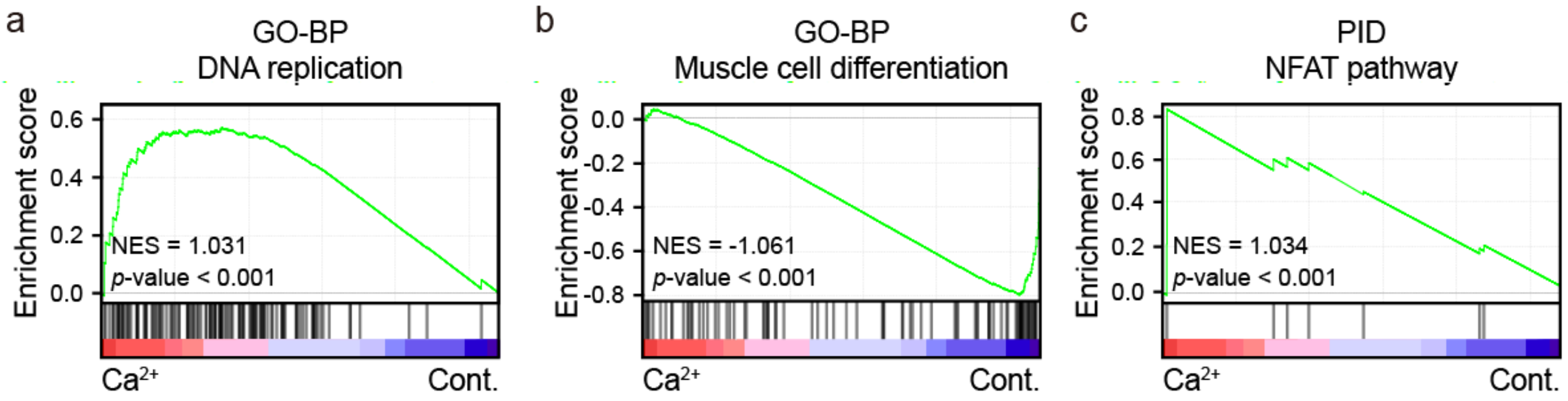


**Supplementary Fig. 3. GSEA enrichment plots of DEGs in $Ca^{2+}$-exposed primary myoblasts.**

GSEA enrichment plots of DEGs for GO-BP DNA replication (**a**), GO-BP muscle cell differentiation (**b**), and PID NFAT TF pathway (**c**). Primary mouse myoblasts were differentiated for 3 days with 0 (Cont.) or 6 mM $CaCl_2$ ($Ca^{2+}$). Normalized Enrichment Scores (NES) and p-values are indicated within each panel.

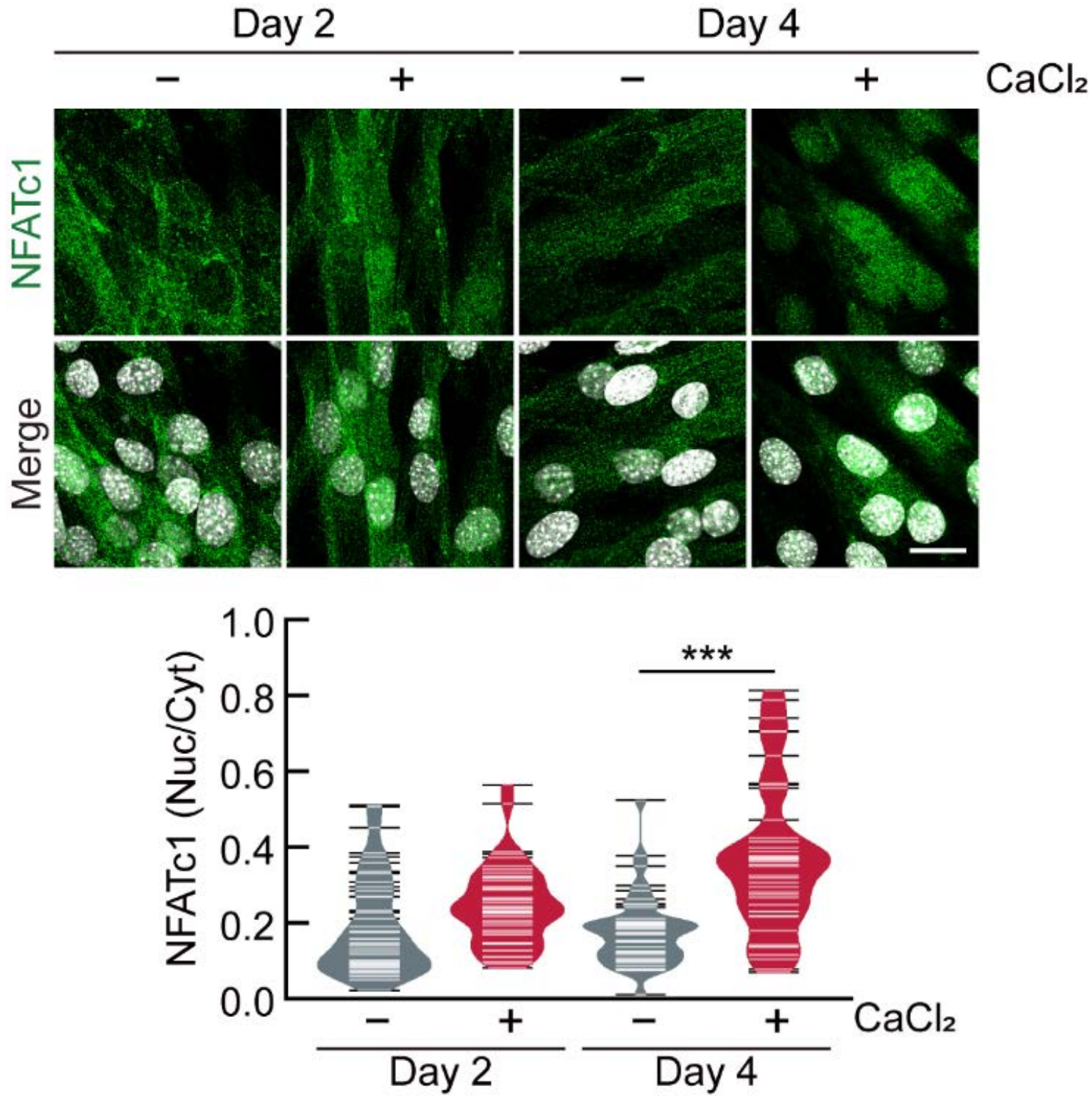


**Supplementary Fig. 4. Excess $Ca^{2+}$ exposure induces NFATc1 nuclear translocation in C2C12 cells.**

Subcellular localization of NFATc1 in C2C12 cells differentiated for 2 or 4 days with 0 or 6 mM $CaCl_2$ and immunostained for NFATc1 (green) and DAPI (white). The nuclear-to-cytoplasmic fluorescence intensity ratio was quantified using ImageJ software. Scale bars, 20 μm. ***$p < 0.001$ using one-way ANOVA followed by Tukey's post hoc test.

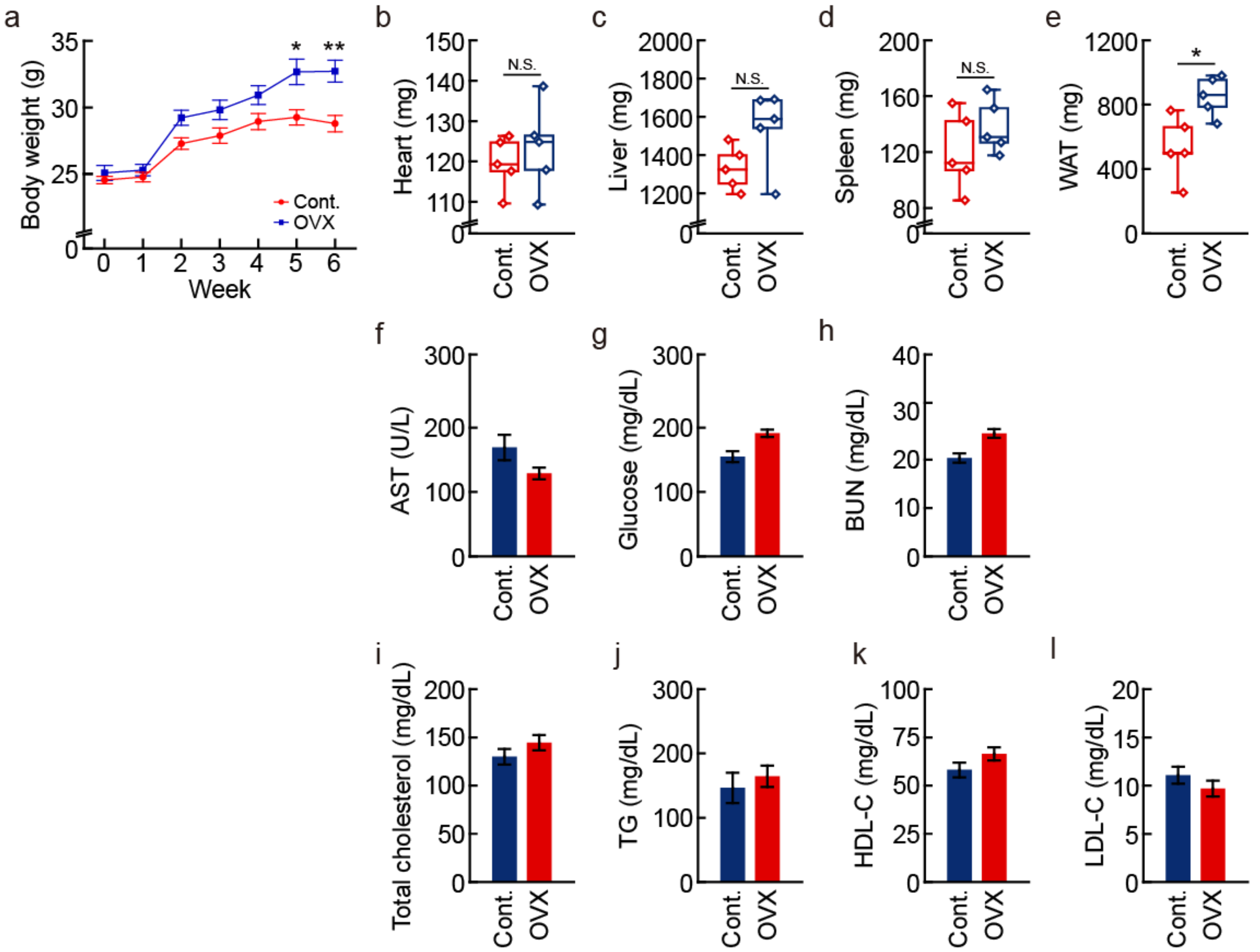


**Supplementary Fig. 5. The physiological parameters of OVX mice.**

**a** Individual body weight of OVX and control mice (n = 5 per group). **b-e** Organ masses of OVX and control mice: heart (**b**), liver (**c**), spleen (**d**) and white adipose tissue (WAT, **e**). Boxes represent the median, with 25th and 75th percentiles (quartiles), and minimum and maximum values. N.S., not significant; *$p < 0.05$ and **$p < 0.01$ using a two-tailed unpaired student's t-test. **f-l** serum biochemical analysis of OVX and control mice: Aspartate aminotransferase (AST, **f**), glucose (**g**), blood urea nitrogen (BUN, **h**), Total cholesterol (**i**), triglycerides (TG, **j**). high-density lipoprotein cholesterol (HDL-C, **k**), and low-density lipoprotein cholesterol (LDL-C, **l**). Data are presented as mean ± SEM.

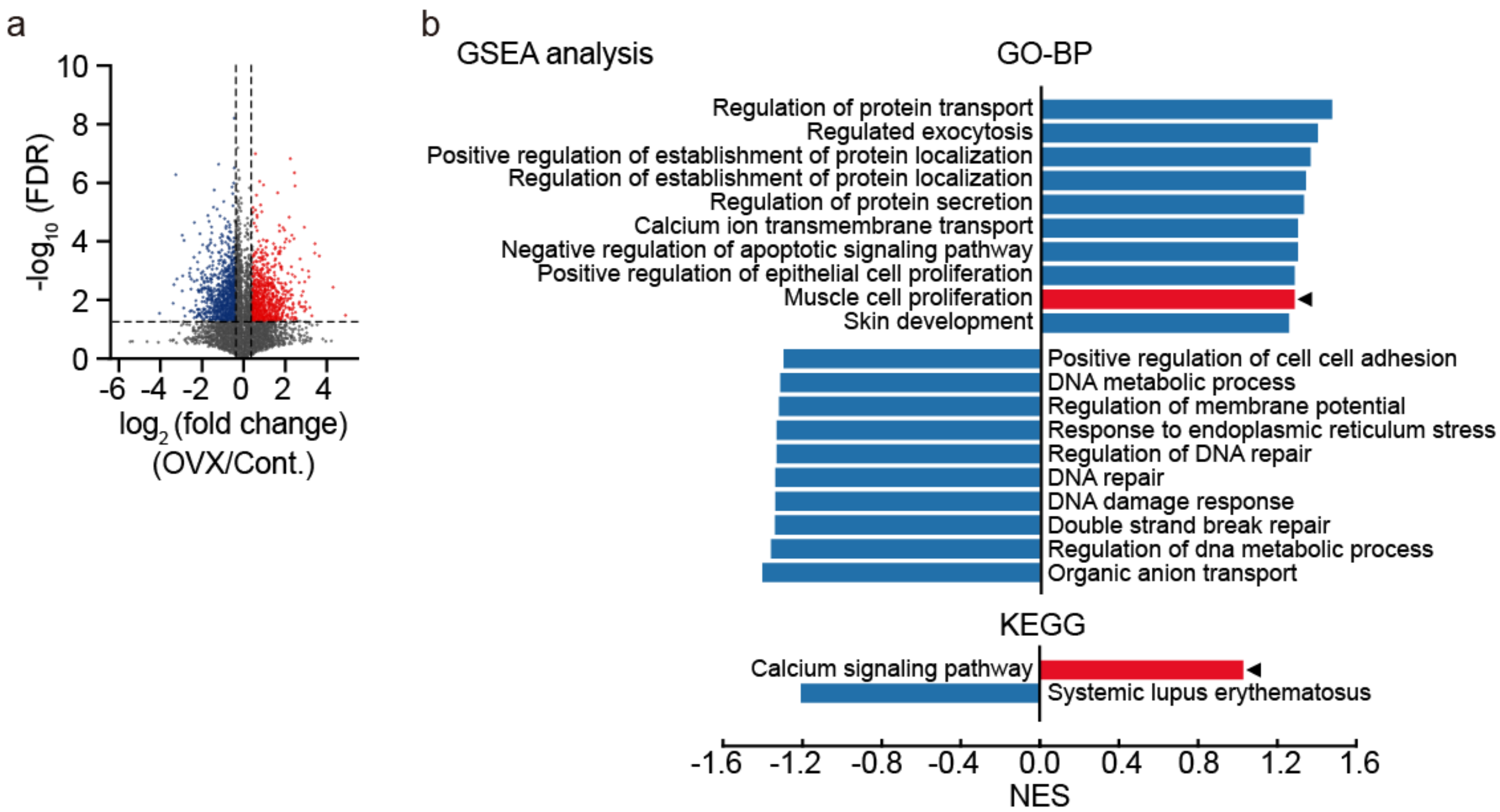


**Supplementary Fig. 6. Transcriptomic profiling of gastrocnemius muscle in OVX mice.**

**a** Volcano plot showing DEGs in gastrocnemius muscle between OVX and control groups (false discovery rate [FDR] < 0.05, fold change > 1.35; n = 2 per group). Red and blue dots represent upregulated and downregulated genes in the OVX group, respectively. **b** GSEA results showing significant enrichments using GO-BP (top) and KEGG (bottom) pathway databases. Gene sets with |NES| > 1, *p*-adjusted < 0.05 were considered significant.

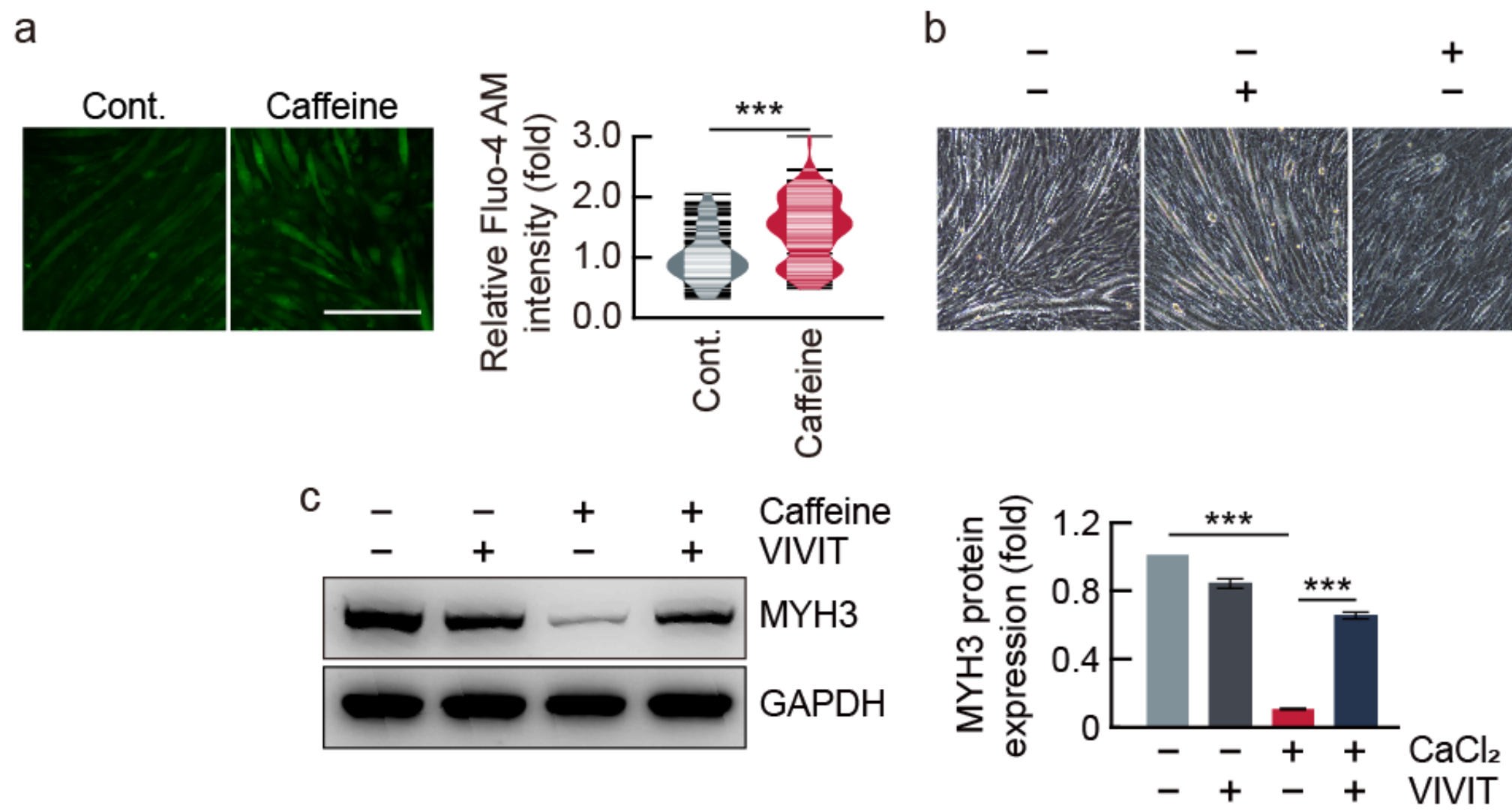


**Supplementary Fig. 7. Caffeine-induced myogenic impairment and its restoration by VIVIT.**

**a** Intracellular $Ca^{2+}$ levels in C2C12 cells after Caffeine exposure. C2C12 cells were differentiated for 5 days with 0, 1 mM caffeine added to the differentiation medium and labeled with the Fluo-4 AM $Ca^{2+}$ indicator. Relative Fluo-4 AM fluorescence intensity was quantified using ImageJ software. Scale bars, 200 μm **b** Representative images of C2C12 cells differentiated for 5 days with 1 mM Caffeine and 20 μM VIVIT, an NFAT inhibitor. Scale bars, 200 μm **c** Immunoblot analysis of MYH3 in C2C12 cells differentiated under indicated conditions. GAPDH served as a loading control. Relative MYH3 expression was quantified. Data are presented as mean ± SEM. ****p* < 0.001 using a one-way ANOVA followed by Tukey's post hoc test.

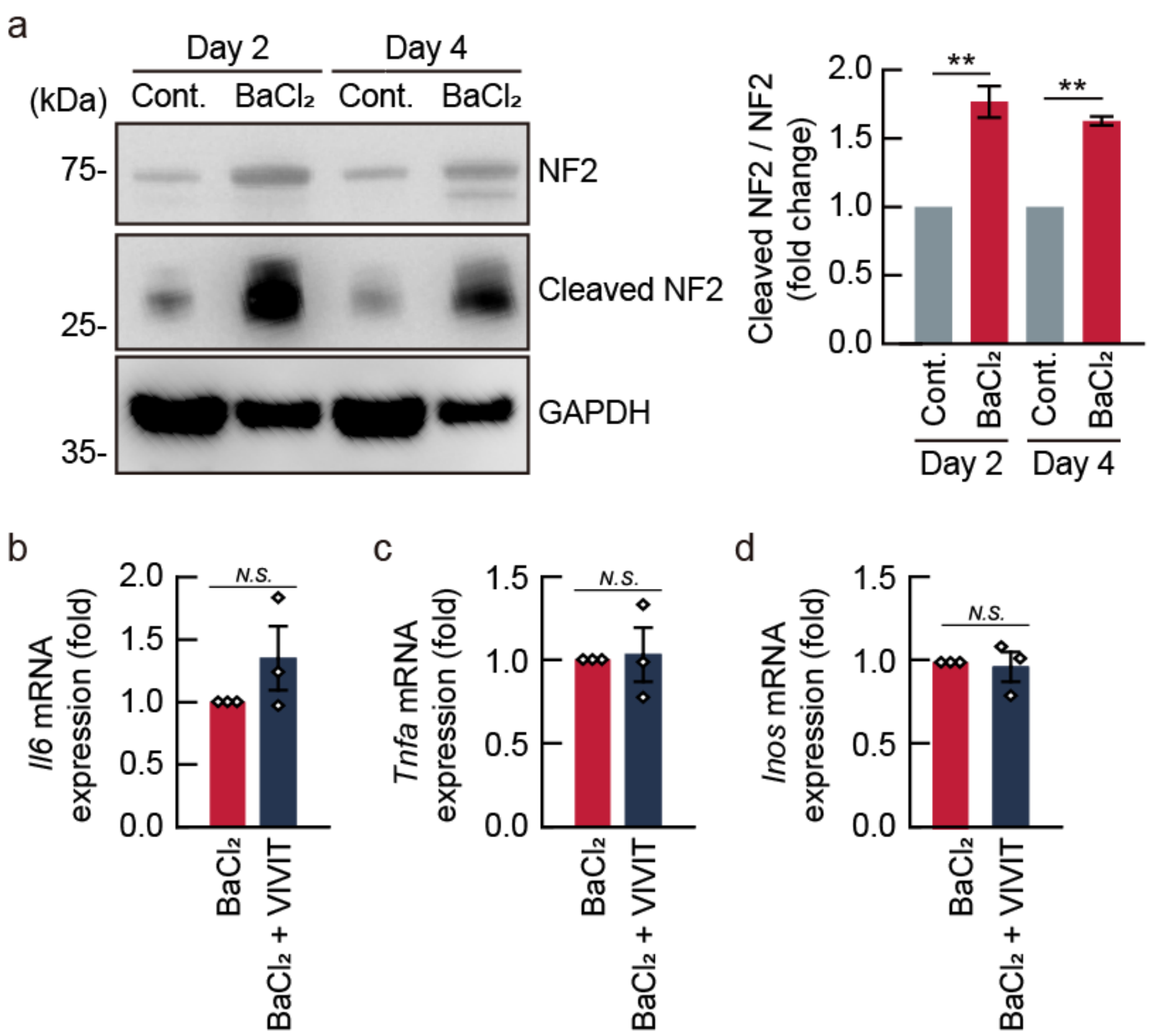


**Supplementary Fig. 8. Activation of $Ca^{2+}$-dependent proteolysis and inflammatory gene expression in the $BaCl_2$-induced muscle injury mouse model.**

**a** Immunoblot analysis of NF2 in tibialis anterior (TA) muscles 2- and 4-days post-injury. GAPDH served as a loading control. Relative cleavage of NF2 was quantified. **b–d** Expression of inflammatory markers *Il6* (**b**), *Tnfa* (**c**), and *Inos* (**d**) in VIVIT-treated muscle compared to those in saline-treated control muscle. 18S rRNA served as an internal control for qRT-PCR. Data are presented as mean ± SEM. N.S., not significant, $^{**}p < 0.01$ using a two-tailed unpaired Student's t-test.

**Supplementary Table 1. Primer sequences for qRT-PCR and ChIP analysis.**

**Supplementary Table 2. Information of GTEx donors.**

**Supplementary Table 3. RNA-seq FPKM values (filtered below 0.01) of calcium-exposed primary mouse myoblasts.**

**Supplementary Table 4. Functional enrichment analysis of $Ca^{2+}$ exposed primary mouse myoblasts.**

**Supplementary Table 5. RNA-seq FPKM values (filtered below 0.01) of gastrocnemius muscle in ovariectomized mice.**

**Supplementary Table 6. Gene Set Enrichment Analysis of gastrocnemius muscle in ovariectomized mice.**
